# NSM-CBAM Planner: A Brain-Inspired Autonomous Collision Avoidance Decision-Making Method for UAVs

**DOI:** 10.3390/biomimetics11100710

**Published:** 2026-10-05

**Authors:** Yongjie Lei, Jianbo Zhou, Lifu Wang

**Affiliations:** 1 College of Mechanical and Electrical Engineering, Northeast Forestry University, Harbin 150040, China; leiyongjie2826@nefu.edu.cn; 2 Harbin Forest Machinery Research Institute, National Forestry and Grassland Administration, Harbin 150086, China

**Keywords:** brain-inspired, collision avoidance, spiking memory CBAM, unmanned aerial vehicle, attention mechanism

## Abstract

Autonomous collision avoidance is one of the key technologies enabling UAVs to successfully carry out measurement tasks in complex and unknown environments. Existing collision avoidance methods typically ignore the spatial and temporal differences in historical information, which makes it hard to achieve effective memory of critical historical states. To address these issues, drawing on high-level bio-inspired principles of spiking neurons and attention, a neuromorphic spiking memory planner (NSM-CBAM) is proposed in this paper. The framework is based on an actor-critic structure, consisting of the spiking memory CBAM actor network (SMCAN) and the conventional fully connected deep critic network (DCN). By introducing spiking memory CBAM into SMCAN, historical information is adaptively invoked in spatial and temporal channels, thereby achieving efficient utilization of historical memory and elimination of redundant historical information. Additionally, soft-reset leaky integrate-and-fire (SR-LIF) neurons are employed to mimic the dynamic changes in membrane potential. Spiking encoders and decoders are employed to convert between continuous states and spiking sequences. Experimental results in a Gazebo/ROS simulation environment show that, compared with other known methods, NSM-CBAM has achieved the highest collision avoidance performance in both unseen evaluation environments, with collision avoidance success rates of up to 94% and 92.5% respectively. This demonstrates the effectiveness and robustness of the proposed framework for autonomous collision avoidance in simulated outdoor environments. Simultaneously, it also provides a promising bio-inspired paradigm with potential computational efficiency for future autonomous collision avoidance of UAVs in more complex environments.

## 1. Introduction

UAV aerial measurement is a novel method currently used in the field of surveying. Compared to traditional measurement techniques, UAV aerial measurement can easily reach locations that are difficult for people to access or explore. In complex, risky, or inaccessible areas and environmental conditions, UAVs can rapidly fly and collect high-quality data, exhibiting flexibility and maneuverability. It plays important roles in fields such as environmental monitoring, urban planning, forestry and other sectors [1,2]. However, UAVs operating in outdoor environments confront unknown, highly complex conditions that raise uncertainty and risk during autonomy and place stringent demands on collision avoidance performance. Consequently, achieving reliable autonomous collision avoidance and intelligent decision-making for UAV in such environments is crucial to improving the safety and efficiency of their operations.

Traditional methods for UAV collision avoidance decision-making mainly include A* [3], the dynamic window approach (DWA) [4,5] and the artificial potential field (APF) [6,7]. Liu et al. [8] integrated A* and APF to produce a hybrid A*-APF method based on spatio-temporal network optimization. This approach improved the performance of the original algorithm and addressed drawbacks such as local minima and failure to reach designated targets. To further improve UAV path planning for collision avoidance, Wang et al. [9] combined four adaptive modules to propose the rapidly converging and greedy-optimized RRT* (RGO-RRT*) algorithm. Compared with traditional RRT*, this method reduced local minima and ensured smooth avoidance trajectories. Zhang et al. [10] presented an improved DWA guided by the sparrow search algorithm (SSA), which adaptively adjusts dynamic window parameters, thereby optimizing the UAV velocity search space and improving flexibility and robustness in local collision avoidance planning relative to traditional DWA. In summary, traditional collision avoidance methods typically rely on prior environmental models or manually designed rules. While they perform adequately in structured settings, their generalization in partially observable environments is limited. These methods often require reconstructing grid maps whenever the environment changes, incurring substantial computational costs. Moreover, although they can avoid collisions, their effectiveness is frequently suboptimal. Rule-based algorithms commonly suffer from entrapment in local minima and a strong dependence on environmental information. They cannot readily improve through experiential learning and therefore lack adaptability and robustness in unfamiliar scenarios, which restricts their suitability for real-time collision avoidance tasks.

Reinforcement learning (RL) enables agents to learn policies through interaction without precise environmental models [11,12,13]. With the development of deep learning, deep reinforcement learning (DRL) [14,15,16] methods such as TD3 [17,18], DDPG [19,20], and PPO [21,22] have been applied to UAV collision avoidance. Variants including MPTD3 [23], two-stream actor–critic [24], PSO-DRL [25], dynamic Q-learning [26], and visual-memory DRL [27] have been proposed to improve convergence and generalization. However, most standard DRL methods rely primarily on the current observation or a Markovian state and do not explicitly retain and exploit historical information. In partially observable and complex environments, this limitation can lead to unstable state estimation and suboptimal collision avoidance decisions.

To address this issue, memory-enhanced DRL methods integrate recurrent or attention-based memory into DRL, such as MATD3 + LSTM [28], PPO + LSTM [29], AG-DQN [30], and GRU-based DRL [31]. These methods enhance the retention and utilization of historical information and improve the ability to capture temporal dependencies. However, most of them embed memory as fixed recurrent layers and process historical information uniformly across spatial and temporal dimensions. They, therefore, cannot adaptively select critical historical states in complex, unstructured environments. In addition, recurrent memory modules increase computational and parameter overhead, which is particularly problematic for resource-constrained UAVs with limited onboard computation and battery capacity.

Spiking neural networks (SNNs) mimic biological spike-based information processing and provide event-driven, low-power spatio-temporal computation [32,33,34]. SNN-based collision avoidance has been studied for mobile robots [35,36]. Wang et al. [37] developed an SNN-based collision avoidance controller that overcomes local-minimum deadlock and enables wall-following navigation. Tang et al. [38] proposed a bio-inspired cognitive navigation system with entorhinal cortex and hippocampal models, in which an SNN stores and retrieves task-relevant information. More recently, SNNs have been combined with DRL for autonomous navigation and collision avoidance [39,40]. These event-driven and low-power characteristics make SNN-based methods well-suited to UAV platforms with limited onboard computation and energy. However, existing SNN + DRL methods still lack adaptive memory selection mechanisms. For example, Zhang et al. [41] proposed a conceptually brain-inspired spiking neural network for underwater robot obstacle avoidance, but its actor network is fully connected and lacks an explicit memory or attention module, so historical information is processed uniformly across time steps. Although some studies combine SNN with LSTM memory structures [42,43,44], they often treat historical information uniformly across space and time and do not explicitly focus on task-critical historical states.

Attention mechanisms can dynamically allocate weights across spatial and temporal dimensions [45,46,47], selectively emphasizing critical historical states. Integrating attention-based memory into SNN + DRL is therefore a promising way to improve the utilization of historical information for UAV collision avoidance. To address this gap, this paper proposes a neuromorphic spiking memory planner (NSM-CBAM) for autonomous UAV collision avoidance. The NSM-CBAM model is built on an actor-critic structure and comprises a spiking memory CBAM actor network (SMCAN) and a conventional fully connected deep critic network (DCN). A spiking memory CBAM module is embedded in the SMCAN to adaptively recall historical information across spatial and temporal channels, thereby using historical memory efficiently while removing redundant information. In addition, the soft-reset leaky integrate-and-fire (SR-LIF) neuron is adopted to mimic, at a high level, the dynamic changes in the membrane potential of biological neurons. Spike encoders and decoders are used for conversion between continuous information and spiking sequences. Comparative experiments have shown that the NSM-CBAM achieves higher success rates than the evaluated baselines in both evaluation environments, while maintaining competitive robustness and generalization. The framework has delivered stable and efficient collision avoidance performance in the evaluated simulated environments and offers a bio-inspired computational approach for future autonomous UAV navigation in more complex environments.

The main contributions of this paper are as follows:

(1) To address autonomous collision avoidance tasks for UAVs in complex and unknown environments, combining SNNs with DRL, the NSM-CBAM model with a spiking memory CBAM is proposed.
(2) The proposed NSM-CBAM consists of SMCAN and DCN. The spiking memory CBAM module is introduced into SMCAN, which adaptively utilizes historical information from spatial and temporal channels to achieve efficient utilization of historical memory and elimination of redundant historical information.
(3) Training results and evaluation results (ablation studies and comparative experiments) have demonstrated that the proposed model achieves improved collision avoidance performance in the evaluated environments.

The remainder of the paper is organized as follows. Section 2 presents the methodological analysis. Section 3 describes the overall framework of NSM-CBAM, including the spiking memory CBAM and algorithm training. Section 4 reports experimental analysis, covering environment setup, training and evaluation performance comparisons, and ablation studies to validate NSM-CBAM. Section 5 concludes and discusses potential directions for future research.

## 2. Methods Analysis

**Figure 1:**
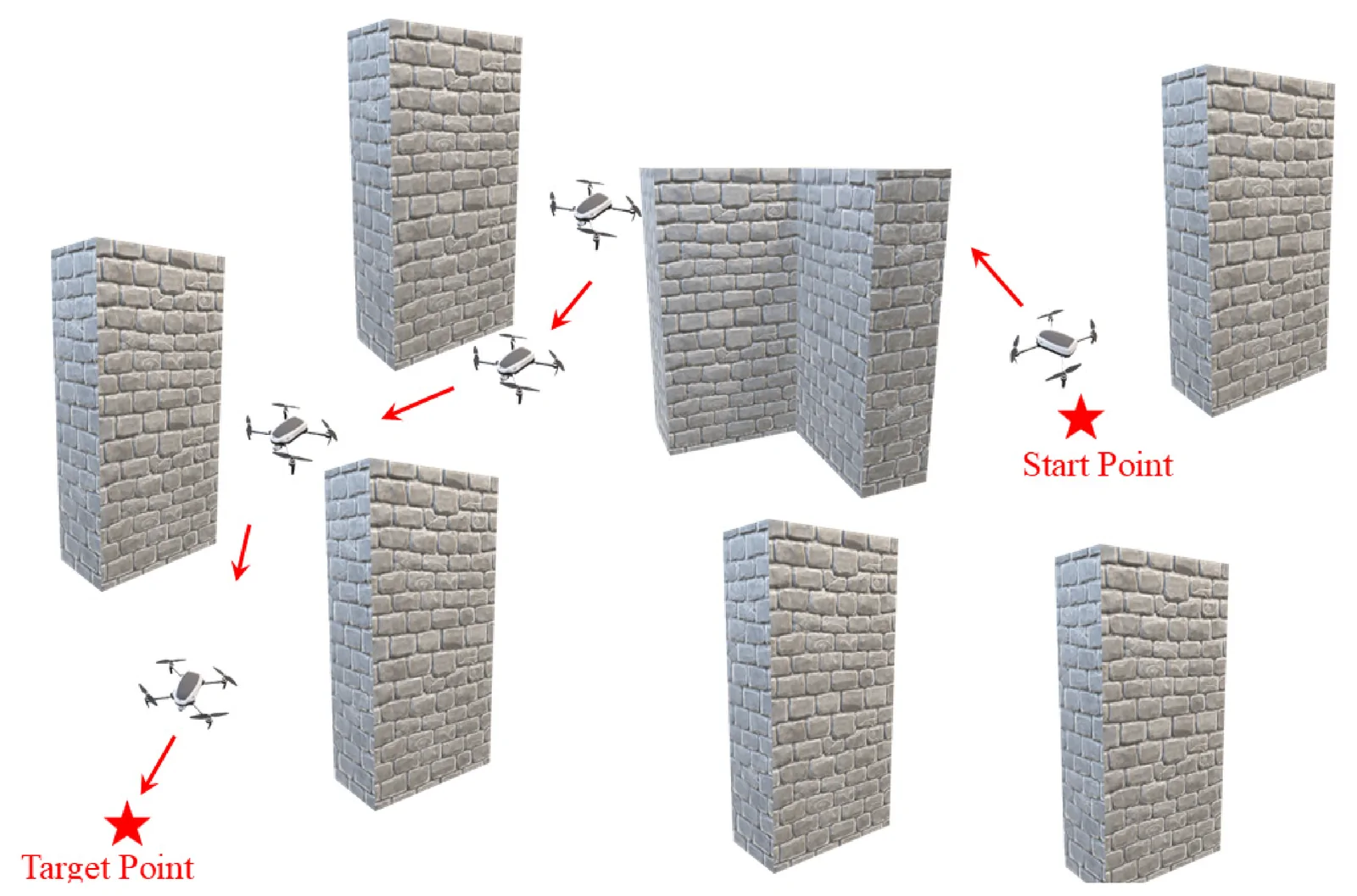
Schematic diagram of UAV collision avoidance.

The autonomous collision avoidance task for a UAV in a complex environment, illustrated in Figure 1, requires real-time decision-making and the generation of control commands using the state estimates and visual observations of the UAV. The objective is to enable the UAV to fly from a randomly assigned start point to a target position when the locations of obstacles in the environment are unknown.

**Figure 2:**
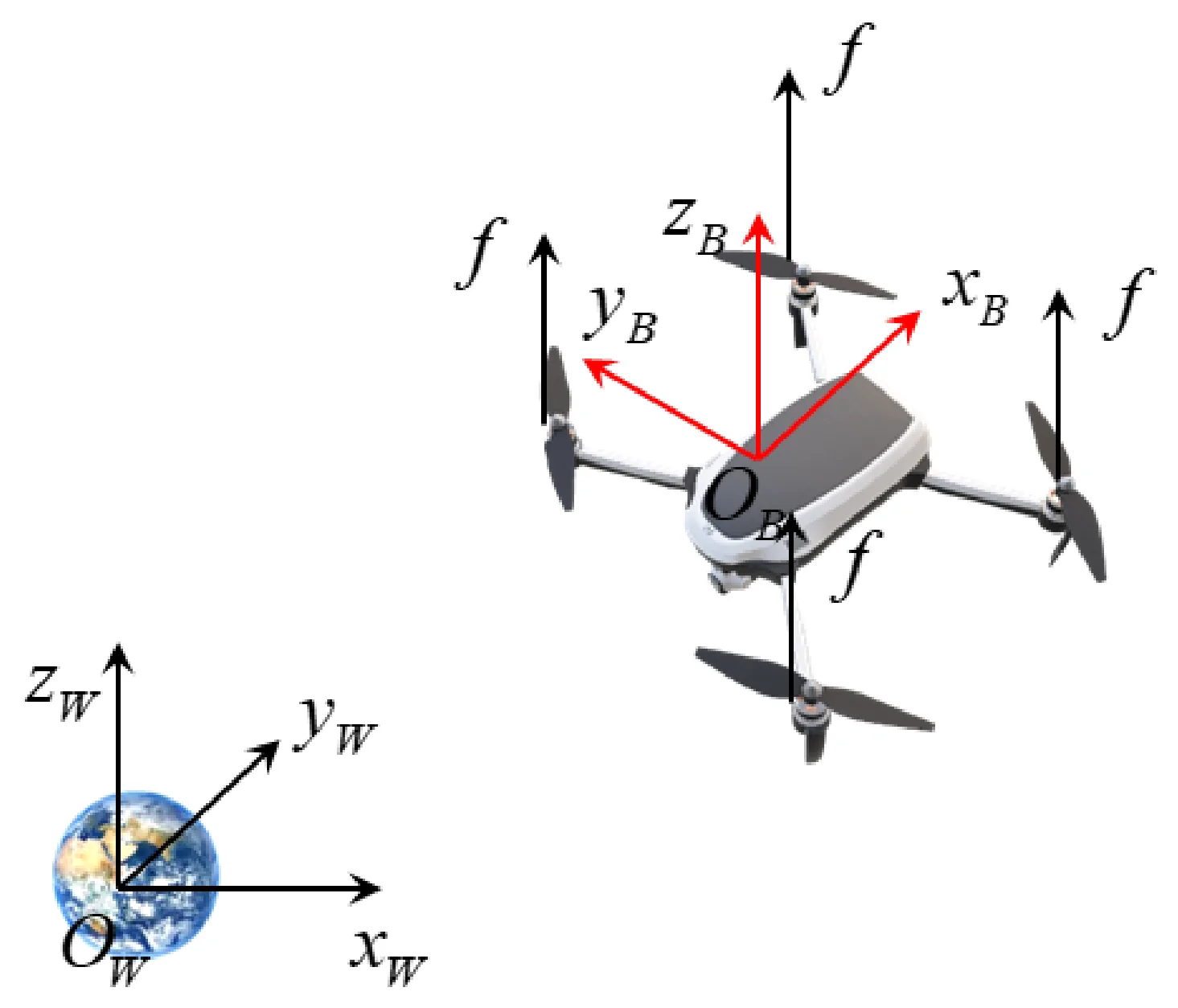
Diagram of the UAV with the world and body frames.

The dynamic model of the UAV is shown in Figure 2. Assuming the UAV is a rigid body, the dynamic equations are expressed as:

(1) $$\dot{x}=\left[\begin{array}{l} \dot{p}\\ \dot{q}\\ \dot{v}\\ \dot{w} \end{array}\right]=\left[\begin{array}{c} v\\ q{\left[0,\omega_{B}^{T}/2\right]}^{T}\\ q⊙f_{T}/m+g\\ I^{-1}\left[\Gamma -\omega \times I\omega \right] \end{array}\right]$$

where *f_T_* represents the thrust, Γ represents the torque, ⊙ represents quaternion multiplication, ***I*** represents the diagonal inertia matrix, *m* represents the mass of the UAV, ***q*** represents the real-time attitude of the UAV, and ***p*** represents the position of the UAV.

### 2.1. Design of State Space and Action Space

The proposed NSM-CBAM is based on an actor-critic network structure. The actor network, composed of spiking neurons, normalizes and spike-encodes the current state. The resulting spike encoding is input to an SMCAN, which produces a spiking sequence representing an action. A spike decoder then converts this sequence into a concrete action, while the critic network evaluates the current state and action. To map the state space to the action space, an SMCAN was incorporated into the NSM-CBAM decision-making framework. The current state is assumed to consist of the relative position between the UAV and the target point, the UAV velocity and visual features from a depth camera.

**Figure 3:**
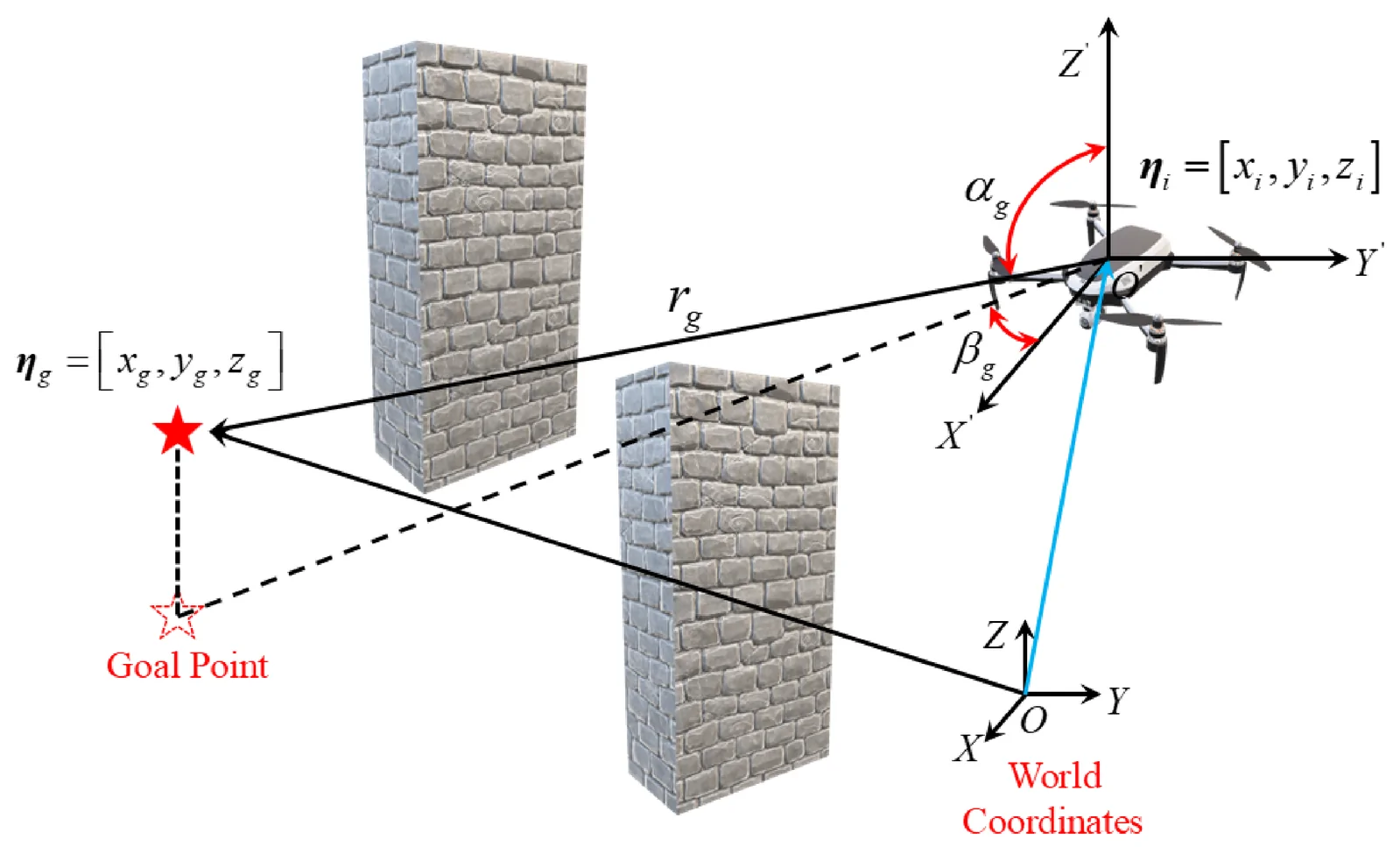
Coordinate system of UAV.

Figure 3 shows the current position of the UAV at a certain moment is ***η****_i_* = [*x_i_*,*y_i_*,*z_i_*], and the target position is ***η****_g_* = [*x_g_*,*y_g_*,*z_g_*]. The local coordinate system is constructed by utilizing the current position of the UAV, and the coordinates of the target point can be expressed as [*r_g_*,*α_g_*,*β_g_*]. Here, *r_g_* represents the radial distance from the UAV to the target, *α_g_* represents the polar angle, and *β_g_* represents the azimuth angle. In the global coordinate system, the linear velocity and angular velocity of the UAV are expressed as *v_i_* and *v_w_*_,*i*_, respectively. Simultaneously, the pooling operation is utilized to divide the depth map ***ω****_i_* into several image patches and calculate the average depth of effective pixels in each patch as visual features. Hence, the UAV state at the i time step is expressed as:

(2) $$S_{i}={\left[r_{i},\alpha_{i},\beta_{i},v_{i},v_{w,i},\omega_{i}\right]}^{T}$$

For the action space, the current action *a_i_* output by the SMCAN is mapped to velocity $\bar{v}=\left[{\bar{v}}_{i},{\bar{v}}_{w,i}\right]$, which is then used to achieve autonomous collision avoidance control of the UAV through velocity control methods.

### 2.2. Spiking Encoder and Spiking Decoder

After normalizing, the current state *S_i_* can be expressed as:

(3) $${\hat{S}}_{i}={\left[{\hat{r}}_{i},{\hat{\alpha }}_{i},{\hat{\beta }}_{i},{\hat{v}}_{i},{\hat{v}}_{w,i},{\hat{\omega }}_{i}\right]}^{T}$$

After normalization, ${\hat{S}}_{i}\in \left[0,1\right]$. For state variables bounded by an upper limit *v_i_* and *α_i_*, conventional scaling is adopted. For positive physical quantities ***ω****_i_* and *r_i_*, they satisfy the inherent lower-bound constraints ***ω****_i_* ≥ ***ω****_min_* > 0 and *r_i_* ≥ *r_min_* > 0 in our UAV simulation. A reciprocal-form normalization is applied such that smaller raw physical values yield larger normalized features, which strengthens the feature response when the UAV is close to the target or operating near the minimum angular velocity. Here, each element is expressed as follows:

(4) $${\hat{v}}_{i}=\frac{v_{i}}{v_{\max}}$$

(5) $${\hat{\omega }}_{i}=\frac{\omega_{\min}}{\omega_{i}}$$

(6) $${\hat{\alpha }}_{i}=\frac{\alpha_{i}}{\pi }$$

(7) $${\hat{r}}_{i}=\frac{r_{\min}}{r_{i}}$$

(8) $${\hat{v}}_{w,i}=\left\{\begin{array}{cc} {\left[\frac{\left|v_{w,i}\right|}{v_{w,\max}},0\right]}^{T} & v_{w,i}\geq 0\\ {\left[0,\frac{\left|v_{w,i}\right|}{v_{w,\max}}\right]}^{T} & v_{w,i}<0 \end{array}\right.$$

(9) $${\hat{\beta }}_{i}=\left\{\begin{array}{cc} {\left[\frac{\left|\beta_{i}\right|}{\pi },0\right]}^{T} & \beta_{i}\geq 0\\ {\left[0,\frac{\left|\beta_{i}\right|}{\pi }\right]}^{T} & \beta_{i}<0 \end{array}\right.$$

After the normalization operation processing, the normalized state ${\hat{S}}_{i}$ is encoded into a set of spiking sequences using the Poisson distribution. The spiking sequence information is expressed as follows:

(10) $$S_{\mathrm{in},i}=\left\{\begin{array}{cc} 1 & \mathrm{if}\;{\hat{S}}_{i}>S_{\mathrm{rand}}\\ 0 & \mathrm{if}\;{\hat{S}}_{i}<S_{\mathrm{rand}} \end{array}\right.$$

where *S_rand_* represents a random number generated from a uniform distribution within [0, 1].

Iterate it *T* times and encode it as a spiking sequence *S_in_*_,*i*_, with time step *T* to serve as the input to the SMCAN, as shown below:

(11) $$S_{\mathrm{in},i}=\mathrm{Spiking}\_\mathrm{Encoder}\left({\hat{S}}_{i}\right)$$

The action output *S_out_*_,*i*_ from the SMCAN appears as a spike train and therefore cannot be applied directly to the UAV autonomous collision avoidance task. A spike decoder converts this output. Specifically, the action spike train is summed and averaged in the time dimension to yield the action spike probability *a_i_*_,*i*_ ∈ [0,1]. This probability is then converted into an executable UAV action *a_i_*. The resulting action is mapped to a velocity command, enabling autonomous collision avoidance by velocity control. The specific formulation is as follows:

*S_out_*_,*i*_ = *y_SMCAN_*((*S_in_*_,*i*_|*δ*) (12)

(13) $$a_{i,j}=\frac{1}{T}\sum_{t=1}^{T}S_{\mathrm{out},i}$$

*a_i_* = *Spiking*_*Decoder*(*S_out_*_,*i*_) (14)

where *y_SMCAN_* denotes the forward propagation mapping function of the proposed SMCAN network, and δ denotes the set of all trainable parameters of SMCAN.

Therefore, during autonomous collision avoidance, the current state of the UAV is processed through the spiking encoder and the spiking decoder to generate the current action, which is then utilized for subsequent network training. The specific expression is as follows:

*a_i_* = *Spiking*_*Decoder*(*y_SMCAN_*(*Spiking*_*Encoder*((*S_in_*|*δ*))) (15)

## 3. Neuromorphic Spiking Memory CBAM

### 3.1. Overall Framework of the Neuromorphic Spiking Memory CBAM

**Figure 4:**
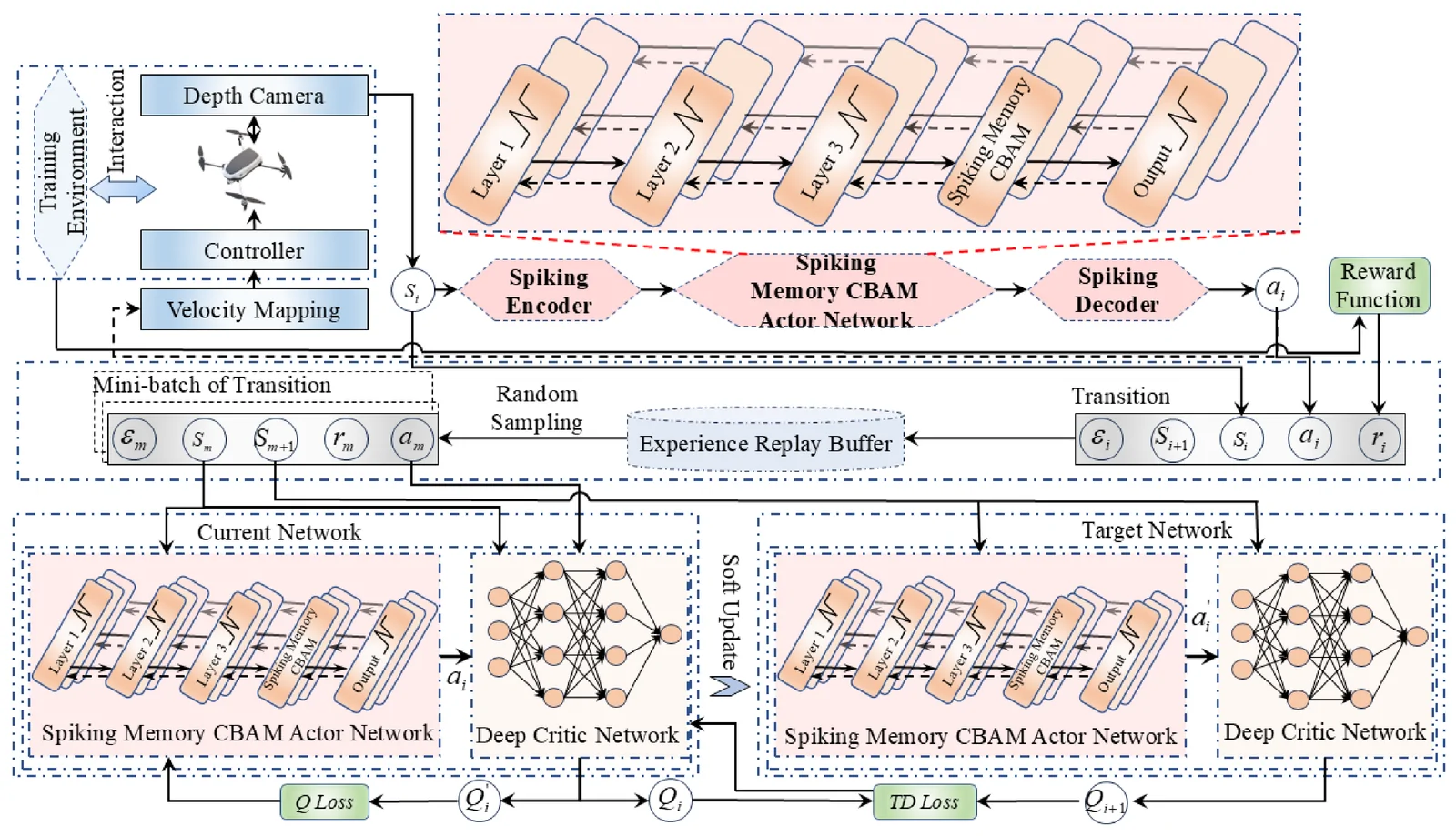
Overall framework diagram of the neuromorphic spiking memory CBAM.

Combining SNN and DRL, spiking memory CBAM with an attention mechanism is introduced, and a NSM-CBAM framework is proposed for the autonomous collision avoidance tasks of UAV in unknown environments. The overall structure of the proposed model is shown in Figure 4. The model consists mainly of SMCAN and DCN. The DCN uses a traditional ANN structure to estimate the action-value function (Q-value), thereby enabling stable policy updates. The SMCAN consists of layer 1, layer 2, layer 3, a spiking memory CBAM module and an output layer. The spiking memory CBAM module combines the temporal modeling capabilities of spiking convolutional LSTM (ConvLSTM) with a spiking CBAM attention mechanism. This design allows the module to capture dynamic changes in historical states across the temporal dimension and adaptively allocate attention weights across channel and spatial dimensions, improving feature extraction and state representation. By better retaining and exploiting historical information, the network makes more informed and robust collision avoidance decisions in complex environments. Furthermore, the core neurons in the SMCAN use SR-LIF units, which provide improved temporal continuity.

The current state *S_i_* is encoded by a spike encoder and supplied as input to the SMCAN. The network uses several hidden layers and the spiking memory CBAM module to efficiently capture spatio-temporal sequence features and historical memory, producing corresponding output information. This output is then decoded into the current action *a_i_* by a spike decoder. A state transition tuple, comprising the current state *S_i_*, current action *a_i_*, next state *S_i_*_+1_, reward value *r_i_*, and the current episode end flag *ε_i_*, is stored in the experience replay buffer. During training, mini-batch transitions are sampled at random from this buffer to update and optimize the network parameters. In addition, target actor and target critic networks are constructed from the SMCAN and the DCN, respectively. The whole architecture is trained jointly by computing the *TD* loss and the *Q* loss to obtain stable policy updates and convergence. The forward propagation of the SMCAN is summarized in Algorithm 1.
**Algorithm 1** Forward Propagation of Spiking Memory CBAM Actor Network *y_SMCAN_*(*δ*)**Require**: Maximum time step *T*; network layers *M*; History State of Spiking Memory CBAM Actor Network *h_o_*.**Input**: Original observation state *S_i_* = [*r_i_*,*α_i_*,*β_i_*,*v_i_*,*v_w_*_,*i*_,*ω_i_*]; weight matrix *w_k_*; bias vector *b_k_*.**Output**: Actual conversion action *a_i_*.1:Through normalization, the original observation state *S_i_* obtains the state spike probability ${\hat{S}}_{i}=\left[{\hat{r}}_{i},{\hat{\alpha }}_{i},{\hat{\beta }}_{i},{\hat{v}}_{i},{\hat{v}}_{w,i},{\hat{\omega }}_{i}\right],$
2:and then sampling and encoding through the Poisson distribution to obtain the spike state sequence *S_in_*_,*i*_ as input to *y_SMCAN_*(*δ*):
$S_{\mathrm{in},i}=\mathrm{Spiking}\_\mathrm{Encoder}\left({\hat{S}}_{i}\right).$
3:Propagate Spiking Memory CBAM Actor Network *y_SMCAN_*(*δ*) with time *t* and layer *k*:4:**for** *t* = 1,⋅⋅⋅,T **do**5:      
$O_{1}^{t}=\mathrm{Layer}1\left(S_{1}^{t}\right)$
6:      
$O_{2}^{t}=\mathrm{Layer}2\left(O_{1}^{t}\right)$
7:      
$O_{3}^{t}=\mathrm{Layer}3\left(O_{2}^{t}\right)$
8:      
$O_{4}^{t},h_{0}=\mathrm{SM}-\mathrm{CBAM}\left(O_{3}^{t},h_{0}\right)$
9:      **for** *m* = 1,⋅⋅⋅,M **do**10:            
$C_{k}^{t}=\eta_{c}\cdot C_{k}^{t-1}+W_{k}\cdot O_{k-1}^{t}+b_{k}$
11:            
$U_{k}^{t}=\eta_{v}\cdot U_{k}^{t-1}-U_{\mathrm{th}}\cdot O_{k}^{t-1}+C_{k}^{t}$
12:            
$O_{k}^{t}=H\left(U_{k}^{t}\right)$
13:
      **end for**
14:      *S_out_*_,*i*_ = *y_SMCAN_*((*S_in_*_,*i*_|*δ*)15:**end for**16:Keep the hidden spike state *h_o_* for the next iteration.17:Decode the output spike *S_out_*_,*i*_ to acquire action spike probability:
$a_{i,i}=\frac{1}{T}\sum_{t=1}^{T}S_{\mathrm{out},i}$

*a_i_* = *Spiking*_*Decoder*(*S_out_*_,*i*_)18:The current action is mapped to a velocity command, enabling autonomous collision avoidance by velocity control.

### 3.2. The SR-LIF Neurons

**Figure 5:**
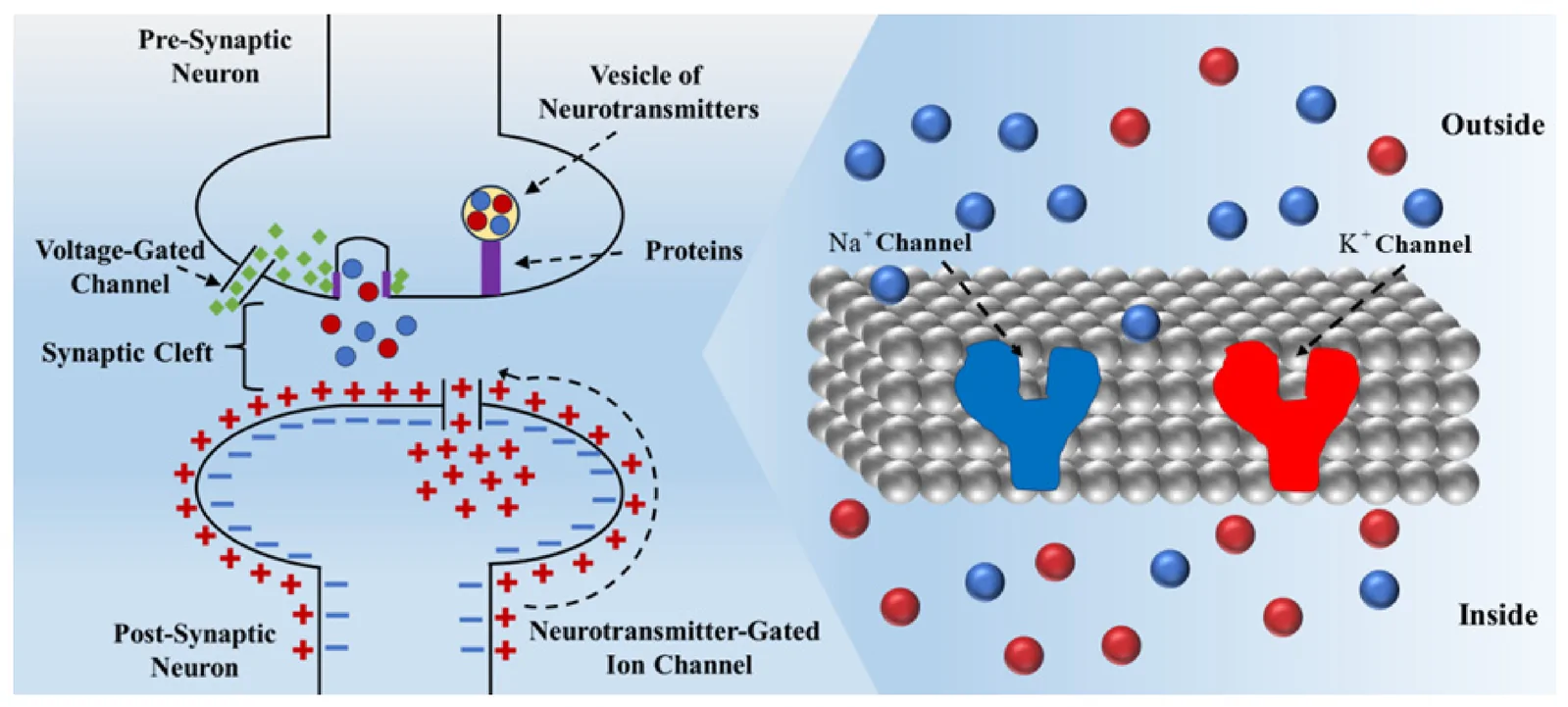
Synaptic structure of the biological neuron [41].

As the fundamental computational unit of SNNs, spiking neurons communicate through spike trains and are conceptually inspired by biological neurons. Their connectivity draws on the general idea of synaptic transmission. These bio-inspired characteristics enable event-driven spatio-temporal computation for engineering tasks. As shown in Figure 5, a neuron receives spike information from other neurons via dendrites. Neurotransmitters are released at the synapses, triggering ion flow through voltage-gated ion channels. When the membrane potential rises and reaches the threshold potential, the neuron generates and fires a spike. This spike is transmitted via the axon to the synaptic terminals of other neurons, facilitating information exchange within the neural network. As the base neuron model, the SR-LIF neuron, in contrast to a hard-reset mechanism, retains the portion of the membrane potential that exceeds the threshold after a spike, rather than resetting directly to the resting potential. By preserving partial historical membrane-potential information, the model maintains smooth and continuous membrane-potential dynamics and reduces excessive firing. Consequently, the SR-LIF neuron provides smoother membrane-potential dynamics and improved temporal continuity. The expression for the SR-LIF neuron is given as follows:

(16) $$C_{k}^{t}=\eta_{c}\cdot C_{k}^{t-1}+W_{k}\cdot O_{k-1}^{t}+b_{k}$$

(17) $$U_{k}^{t}=\eta_{v}\cdot U_{k}^{t-1}-U_{\mathrm{th}}\cdot O_{k}^{t-1}+C_{k}^{t}$$

(18) $$O_{k}^{t}=H\left(U_{k}^{t}\right)$$

where $C_{k}^{t}$ represents the membrane current, $U_{k}^{t}$ is the membrane voltage, *η_c_* and *η_v_* are the decay factors for the current and voltage, respectively, *W_k_* is the synaptic weight matrix, *b_k_* is the synaptic bias vector, $H\left(U_{k}^{t}\right)$ is the indicator function, and *U*_th_ is the membrane potential threshold.

### 3.3. Spiking Memory CBAM

**Figure 6:**
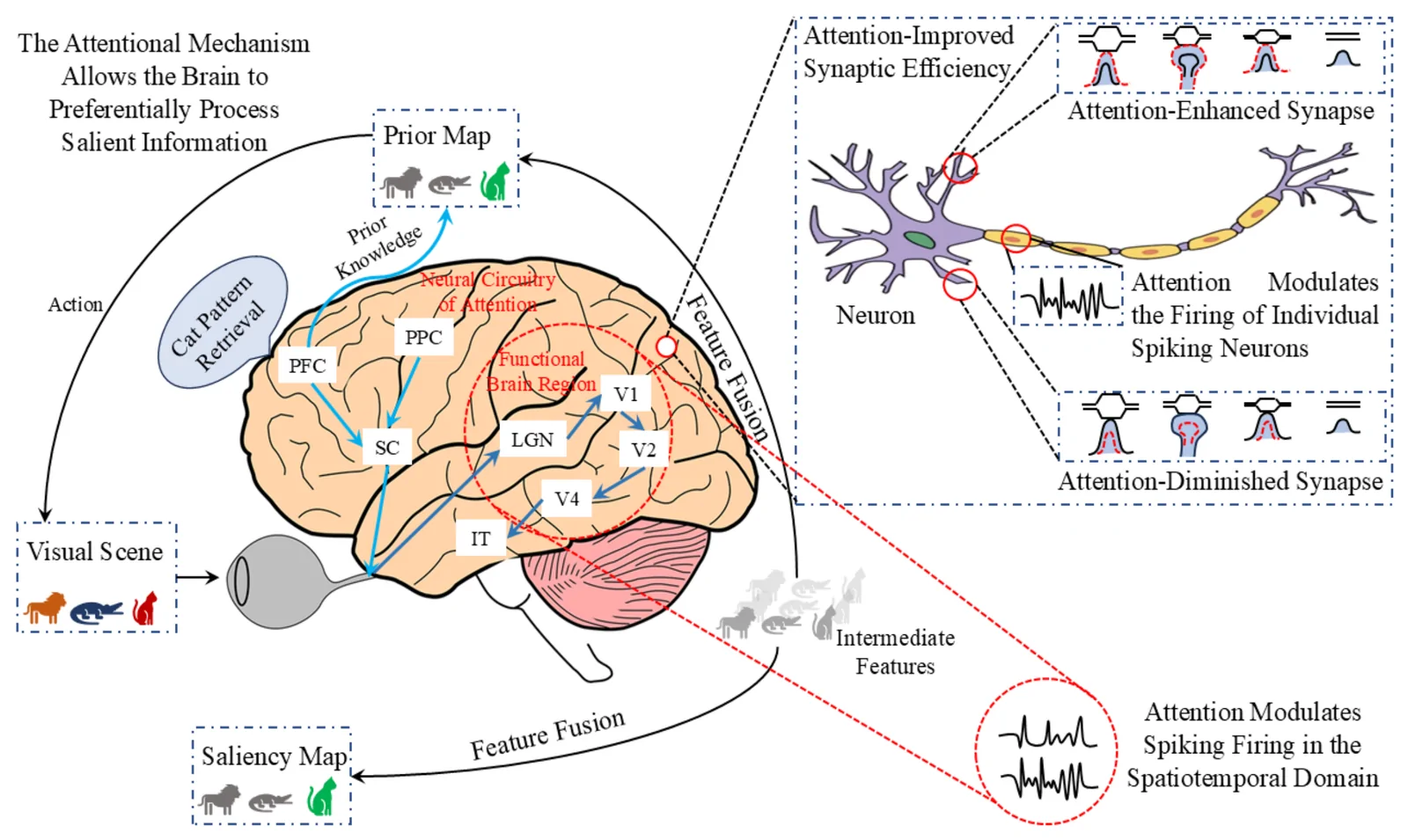
Attention mechanisms in the brain across multiple hierarchical levels.

**Figure 7:**
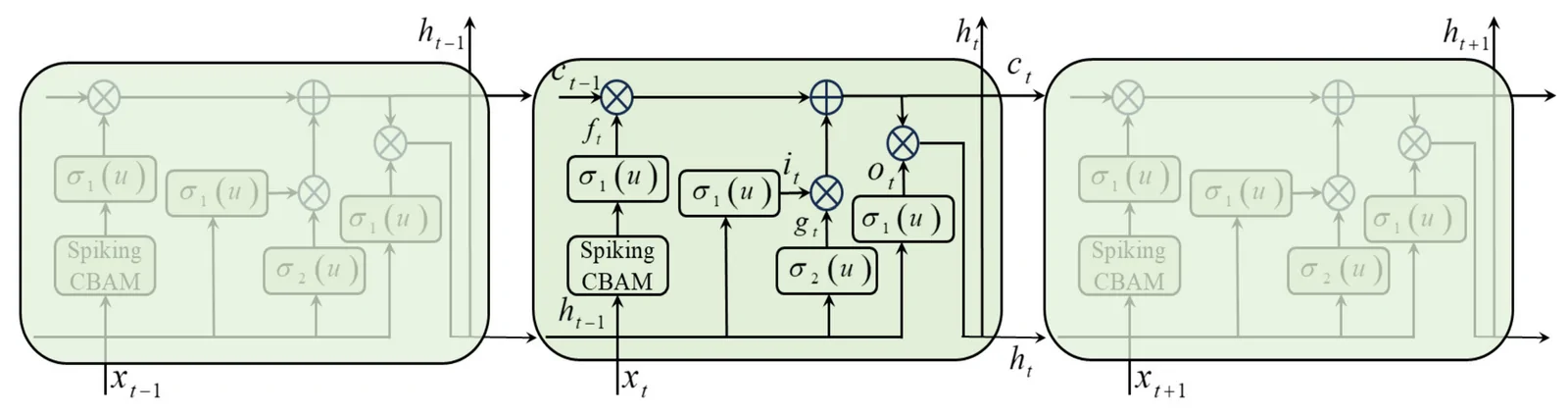
Spiking memory CBAM cell.

The attentional mechanism (as shown in Figure 6) can enable the human brain to selectively focus on task-relevant key information during decision-making, thereby efficiently extracting significant features in complex environments. Inspired by the attention mechanism of the human brain, the spiking memory CBAM module with a spiking attention mechanism is proposed. The proposed spiking memory CBAM is shown in Figure 7. The module utilizes spiking CBAM to modify the forget gate of spiking ConvLSTM, which focuses on important historical information in both spatial and channel dimensions. Spiking memory CBAM can adaptively invoke historical information from spatial and temporal channels based on spatial and temporal features, which enhances its utilization of critical historical information, as expressed by the following equation:

(19) $$f_{l}^{\left(t\right)}=\sigma_{1}(w_{f,h}*h_{l-1}^{\left(t\right)}+w_{f,x}*x_{l}^{\left(t\right)}+b_{f})$$

(20) $$i_{l}^{\left(t\right)}=\sigma_{1}(w_{i,h}*h_{l-1}^{\left(t\right)}+w_{i,x}*x_{l}^{\left(t\right)}+b_{i})$$

(21) $$g_{l}^{\left(t\right)}=\sigma_{2}(w_{g,h}*h_{l-1}^{\left(t\right)}+w_{g,x}*x_{l}^{\left(t\right)}+b_{g})$$

(22) $$c_{t}^{\left(t\right)}=f_{l}^{\left(t\right)}⊙c_{l-1}^{\left(t\right)}+i_{l}^{\left(t\right)}⊙g_{l}^{\left(t\right)}$$

(23) $$o_{l}^{\left(t\right)}=\sigma_{1}(w_{o,h}*h_{l-1}^{\left(t\right)}+w_{o,x}*x_{l}^{\left(t\right)}+b_{o})$$

(24) $$h_{l}^{\left(t\right)}=o_{l}^{\left(t\right)}⊙c_{l}^{\left(t\right)}$$

where ∗ and ⊙ represent convolution operation and Hadamard product, *f* represents the forget gate, *i* represents the input gate, o represents the output gate, *c* represents the inner state, *h* represents the output state, *g* represents the candidate cell state, *x* is the input, while *w* and *b* represent the weight and bias parameters, respectively. Furthermore, in the traditional ConvLSTM, activation functions such as sigmoid and tanh are employed to map data, enabling selective information transmission and updating. Likewise, in the spiking memory CBAM, the activation functions *σ*_1_ and *σ*_2_ assess whether the membrane potential exceeds the firing threshold. When it does, a spike is generated, and the potential is reset, producing sparse encodings and dynamic spatio-temporal feature updates.

### 3.4. Spiking Memory CBAM

**Figure 8:**
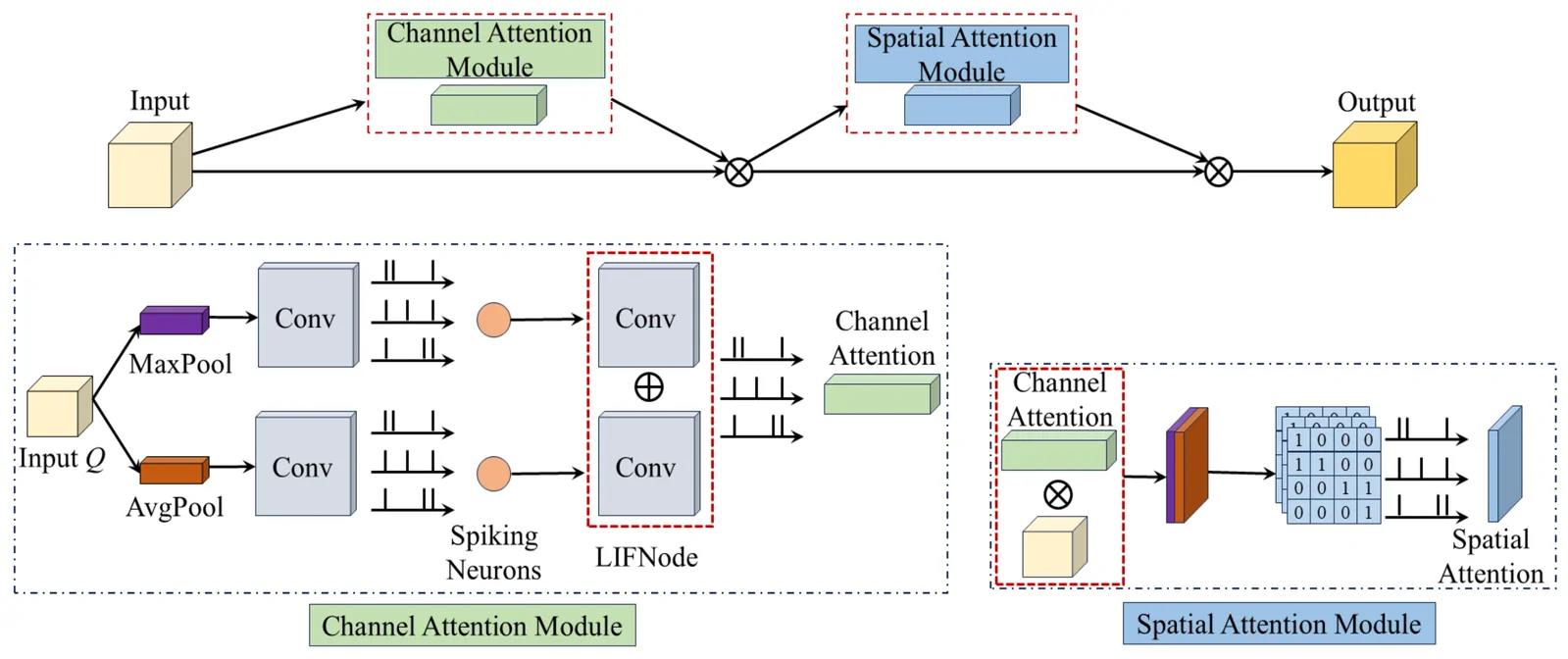
Spiking CBAM module.

The spiking CBAM is illustrated in Figure 8. It consists of the channel attention mechanism and the spatial attention mechanism, which are expressed as follows:

*Q*^′^ = *M_C_*(*Q*)⊗*Q* (25)

*Q*^″^ = *M_S_*(*Q*^′^)⊗*Q*^′^ (26)

where *Q* denotes the input feature tensor fed into the spiking CBAM module, *M_C_* represents the channel attention module, and *M_S_* represents the spatial attention module. The operations *M_C_* and *M_S_* sequentially perform channel information and spatial information extraction, generating the channel attention module *M_C_*(*Q*) and the spatial attention module *M_S_*(*Q*^′^), respectively.

The channel attention module employs both average pooling and max pooling operations and generates channel attention information through a spiking-MLP. The expression is as follows:

*M_C_*(*Q*) = *σ*(*SMLP*(*AvgPool*(*Q*)) + *SMLP*(*MaxPool*(*Q*))) (27)

where *σ* denotes the spiking activation function, *SMLP*(·) denotes the spiking multi-layer perceptron, and *AvgPool*(·) and *MaxPool*(·) represent global average pooling and global maximum pooling operations, respectively.

The spatial attention module employs average pooling and max pooling operations, followed by a convolution operation. The resulting information is then passed through a spiking activation function to generate spatial attention information. The expression is as follows:

*M_S_*(*Q*^′^) = *σ*(*f*^7×7^([*AvgPool*(*Q*^′^);*MaxPool*(*Q*^′^)])) (28)

where *σ* denotes the spiking activation function, and *f*^7×7^ represents a convolution operation with the filter size of 7 × 7.

### 3.5. Algorithm Training

During the training process, it is necessary to determine whether the UAV can safely clear an obstacle. The UAV is constrained to fly at a fixed altitude during training, and the planar distance between the obstacle and the UAV within the same horizontal plane is computed. If this distance is smaller than a predefined collision threshold, a collision is deemed to have occurred; otherwise, the UAV is considered to have safely passed the obstacle.

The detailed training procedure of the NSM-CBAM is outlined in Algorithm 2. During the autonomous collision avoidance training process, the UAV utilizes the SMCAN *y_SMCAN_*(*δ*) to generate the current action *a_i_* based on the present state *S_i_*, thereby executing an avoidance behavior. As the UAV interacts with the environment, the predefined reward function yields an immediate reward for each time step. Furthermore, each training step produces a transition tuple, which is stored in the experience replay buffer. Mini-batch transitions [*S_i_*,*S_i_*_+1_,*a_i_*,*r_i_*,*ε_i_*] are subsequently sampled randomly from this buffer to facilitate network training. The NSM-CBAM establishes connections between *y_SMCAN_*(*δ*), *y_DCN_*(*ο*) and their corresponding target networks *y_TSMCAN_*(*δ*^′^) and *y_TDCN_*(*ο*^′^). Finally, the overall network is trained using the *TD* Loss and *Q* Loss, with the specific calculations provided as follows:

(29) $$\begin{array}{cc} L_{\mathrm{TD}}\left(ο\right)= & {\left(r_{i}+\gamma Q_{i+1}-Q_{i}\right)}^{2}\\ = & {\left(\begin{array}{l} r_{i}+\gamma y_{\mathrm{DCN}}\left(S_{i+1},\mathrm{Spiking}\_\mathrm{Decoder}\left(y_{\mathrm{SMCAN}}\left(\mathrm{Spiking}\_\mathrm{Encoder}\left(S_{i+1}\right)|\delta^{\prime }\right)\right){ο}^{\prime }\right)\\ -y_{\mathrm{DCN}}\left(S_{i},\mathrm{Spiking}\_\mathrm{Decoder}\left(y_{\mathrm{SMCAN}}\left(\mathrm{Spiking}\_\mathrm{Encoder}\left(S_{i}\right)|\delta \right)\right)|ο\right) \end{array}\right)}^{2} \end{array}$$

(30) $$\begin{array}{cc} L_{Q}\left(\delta \right)= & -Q_{i}\\ = & -y_{\mathrm{DCN}}\left(S_{i},\mathrm{Spiking}\_\mathrm{Decoder}\left(y_{\mathrm{SMCAN}}\left(\mathrm{Spiking}\_\mathrm{Encoder}\left(S_{i}\right)|\delta \right)\right)|ο\right) \end{array}$$

where *y_DCN_*(·) denotes the forward mapping function of the DCN Q-network, *y_TDCN_*(·) represents its corresponding target network, δ is the set of trainable parameters of SMCAN, *o* and *o’* denote the observation input at current step and next step, respectively, γ is the discount factor for reinforcement learning, *L_TD_*(*ο*) is the temporal-difference loss, and *L_Q_*(*δ*) is the loss function for updating SMCAN parameters.

The role of the reward function during training is to guide the UAV in successfully avoiding obstacles. The expression is as follows:

(31) $$r_{o}=\left\{\begin{array}{cc} r_{\mathrm{goal}} & \mathrm{if}\;\;\;\;D_{\mathrm{dis}}\left(P_{o},P_{\mathrm{goal}}\right)<D_{\mathrm{goal}\_\mathrm{th}}\\ r_{\mathrm{obs}} & \mathrm{if}\;\;\;\;D_{\mathrm{dis}}\left(P_{o},P_{\mathrm{obs}}\right)<D_{\mathrm{obs}\_\mathrm{th}}\\ r_{\mathrm{other}} & \;\;\;\;\;\;\;\mathrm{otherwise} \end{array}\right.$$

*r_other_* = *α* ⋅ (*D_dis_*(*P_o_*,*P_goal_*) − *D_dis_*(*P_o_*_−1_,*P_goal_*)) (32)

where *r_goal_* and *r_obs_* represent the reward values for successfully reaching the target point and for colliding with an obstacle, respectively. *D_dis_*(*P_o_*,*P_goal_*) represents the distance from the UAV to the target point. If *D_dis_*(*P_o_*,*P_goal_*) is less than a predefined threshold hyperparameter *D_goal_*__*th*_, the UAV is considered to have reached the target. *D_dis_*(*P_o_*,*P_obs_*) represents the distance from the UAV to the obstacle. If *D_dis_*(*P_o_*,*P_obs_*) is less than a collision threshold *D_obs_*__*th*_, a collision is registered. *r_other_* = *α* ⋅ (*D_dis_*(*P_o_*,*P_goal_*) − *D_dis_*(*P_o_*_−1_,*P_goal_*)) represents the change in the distance to the target between the current time step and the previous one.

To enhance the stability of the learning process, the soft update mechanism is employed to update the parameters of the target networks *y_TSMCAN_*(*δ*^′^) and *y_TDCNN_*(*ο*^′^). The expression is as follows:

*δ*^′^ = *ηδ* + (1 − *η*)*δ*^′^_target_ (33)

*ο*^′^ = *ηο* + (1 − *η*)*ο*^′^_target_ (34)

where *δ* and *δ*^′^_target_ denote the parameter sets of the main SMCAN network and its corresponding target network, respectively; *ο* and *ο*^′^_target_ represent the parameter sets of the main DCN critic network and its target TDCN network; and *η* is the soft-update coefficient for target-network parameter smoothing.
**Algorithm 2** Specific Flow of NSM-CBAM.**Input:** Spiking memory CBAM actor network *y_SMCAN_*(*δ*); deep critic normal network *y_DCNN_*(*ο*); corresponding target network *y_TSMCAN_*(*δ*^′^) and *y_TDCNN_*(*ο*^′^).**Output**: *TD* loss *L_TD_*(*ο*); *Q* loss *L_Q_*(*δ*).1:Randomly initialize spiking memory CBAM actor network *y_SMCAN_*(*δ*) and deep critic normal network *y_DCNN_*(*ο*) with random parameters *δ* and *ο*, respectively.2:Initialize the corresponding target network *y_TSMCAN_*(*δ*^′^) and *y_TDCNN_*(*ο*^′^) respectively by copying the same parameters *δ*^′^ ← *δ* and *ο*^′^ ← *ο*.3:Initialize experience replay buffer *E.*4:**for** *x* = 1,⋅⋅⋅,*X* **do**5:      Obtain the original state *S_i_* of the UAV environment.6:      **for** *o* = 1,⋅⋅⋅,*O* **do**7:      Obtain action *a_i_* = *Spiking*_*Decoder*(*y_SMCAN_*(*Spiking*_*Encoder*((*S_in_*|*δ*))) + *Ni*, where *N_i_*~OU(*μ, θ, σ*), and clip *a_i_* to the valid action range.8:      Execute action *a_i_* and get the reward *r_i_*, so that the new environment state changes to *S_i_*_+1_.9:      Store [*S_i_*,*S_i_*_+1_,*a_i_*,*r_i_*,*ε_i_*] as a transition into the experience replay buffer *E*.10:      Random sampling of mini-batch transitions from experience replay buffer *E* for hybrid training.11:      Update the critic network by minimizing *L_TD_*(*ο*):            $\begin{array}{cc} L_{\mathrm{TD}}\left(ο\right)= & {\left(r_{i}+\gamma Q_{i+1}-Q_{i}\right)}^{2}\\ = & {\left(\begin{array}{l} r_{i}+\gamma y_{\mathrm{DCNN}}\left(S_{i+1},\mathrm{Spiking}\_\mathrm{Decoder}\left(y_{\mathrm{SMCAN}}\left(\mathrm{Spiking}\_\mathrm{Encoder}\left(S_{i+1}\right)|\delta^{\prime }\right)\right){ο}^{\prime }\right)\\ -y_{\mathrm{DCNN}}\left(S_{i},\mathrm{Spiking}\_\mathrm{Decoder}\left(y_{\mathrm{SMCAN}}\left(\mathrm{Spiking}\_\mathrm{Encoder}\left(S_{i}\right)|\delta \right)\right)|ο\right) \end{array}\right)}^{2} \end{array}$
12:            Update the actor network by maximizing *L_Q_*(*δ*):            $\begin{array}{cc} L_{Q}\left(\delta \right)= & -Q_{i}\\ = & -y_{\mathrm{DCNN}}\left(S_{i},\mathrm{Spiking}\_\mathrm{Decoder}\left(y_{\mathrm{SMCAN}}\left(\mathrm{Spiking}\_\mathrm{Encoder}\left(S_{i}\right)|\delta \right)\right)|ο\right) \end{array}$
13:            Soft-update the target network *y_TSMCAN_*(*δ*^′^) and *y_TDCNN_*(*ο*^′^):            *δ*^′^ = *ηδ* + (1 − *η*)*δ*^′^*target*            *ο*^′^ = *ηο* + (1 − *η*)*ο*^′^*target*14:
      **end for**
15:**end for**

During training, the spatio-temporal backpropagation (STBP) algorithm [48] is used to carry out policy-gradient backpropagation for the proposed NSM-CBAM. Due to the non-differentiable property of SNNs, a rectangular function is adopted as the pseudo-gradient to approximate the gradient of spike generation. Here, *λ* is a non-zero constant and *U*_th_ denotes the spike firing threshold. The policy-gradient backpropagation using STBP is computed as follows:

(35) $$T\left(U_{k}^{t}\right)=\frac{1}{\lambda }G\left(\left|U_{k}^{t}-U_{\mathrm{th}}\right|-\frac{\lambda }{2}\right)$$

where *G*(·) denotes the rectangular-window function. It outputs 1 if its argument lies within the specified window, and 0 otherwise.

The backpropagation process is differentiated based on time step t and network layer *n*. |s denotes the derivative in the spatial domain and |t denotes the derivative in the temporal domain. The expression is as follows:

(36) $$\frac{\partial L_{Q}\left(\delta \right)}{\partial O_{M}^{T}}=\left\{\begin{array}{c} \frac{\partial L_{Q}\left(\delta \right)}{\partial O_{M}^{T}}\left|s\right.=\frac{1}{T}\cdot \frac{\partial L_{Q}\left(\delta \right)}{\partial a},\;\;\;t=T,m=M\\ \frac{\partial L_{Q}\left(\delta \right)}{\partial O_{m}^{T}}\left|s\right.=W_{m+1}\cdot \frac{\partial L_{Q}\left(\delta \right)}{\partial C_{m+1}^{T}},\;\;\;t=T,m<M\\ \frac{\partial L_{Q}\left(\delta \right)}{\partial O_{M}^{t}}\left|t\right.=\frac{\partial L_{Q}\left(\delta \right)}{\partial U_{M}^{t+1}}\cdot \left(-U_{\mathrm{th}}\right),\;\;\;t<T,m=M\\ \frac{\partial L_{Q}\left(\delta \right)}{\partial O_{m}^{t}}\left|s\right.+\frac{\partial L_{Q}\left(\delta \right)}{\partial O_{m}^{t}}\left|t\right.=W_{m+1}\cdot \frac{\partial L_{Q}\left(\delta \right)}{\partial C_{m+1}^{t}}+\frac{\partial L_{Q}\left(\delta \right)}{\partial U_{m}^{t+1}}\cdot \left(-U_{\mathrm{th}}\right),\;\;\;t<T,m<M \end{array}\right.$$

(37) $$\frac{\partial L_{Q}\left(\delta \right)}{\partial U_{M}^{T}}=\left\{\begin{array}{c} \frac{\partial L_{Q}\left(\delta \right)}{\partial U_{M}^{T}}\left|s\right.=\dot{T}\left(U_{M}^{T}\right)\cdot \frac{\partial L_{Q}\left(\delta \right)}{\partial O_{M}^{T}},\;\;\;t=T,m=M\\ \frac{\partial L_{Q}\left(\delta \right)}{\partial U_{m}^{T}}\left|s\right.=\dot{T}\left(U_{m}^{T}\right)\cdot \frac{\partial L_{Q}\left(\delta \right)}{\partial O_{m}^{T}},\;\;\;t=T,m<M\\ \frac{\partial L_{Q}\left(\delta \right)}{\partial U_{M}^{t}}\left|t\right.=\eta_{v}\cdot \frac{\partial L_{Q}\left(\delta \right)}{\partial U_{M}^{t+1}},\;\;\;t<T,m=M\\ \frac{\partial L_{Q}\left(\delta \right)}{\partial U_{m}^{t}}\left|s\right.+\frac{\partial L_{Q}\left(\delta \right)}{\partial U_{m}^{t}}\left|t\right.=\dot{T}\left(U_{m}^{t}\right)\cdot \frac{\partial L_{Q}\left(\delta \right)}{\partial O_{m}^{t}}+\eta_{v}\cdot \frac{\partial L_{Q}\left(\delta \right)}{\partial U_{m}^{t+1}},\;\;\;t<T,m<M \end{array}\right.$$

(38) $$\frac{\partial L_{Q}\left(\delta \right)}{\partial C_{M}^{T}}=\left\{\begin{array}{c} \frac{\partial L_{Q}\left(\delta \right)}{\partial C_{M}^{T}}\left|s\right.=\frac{\partial L_{Q}\left(\delta \right)}{\partial U_{M}^{T}},\;\;\;t=T,m=M\\ \frac{\partial L_{Q}\left(\delta \right)}{\partial C_{m}^{T}}\left|s\right.=\frac{\partial L_{Q}\left(\delta \right)}{\partial U_{m}^{T}},\;\;\;t=T,m<M\\ \frac{\partial L_{Q}\left(\delta \right)}{\partial C_{M}^{t}}\left|t\right.=\eta_{c}\cdot \frac{\partial L_{Q}\left(\delta \right)}{\partial C_{M}^{t+1}},\;\;\;t<T,m=M\\ \frac{\partial L_{Q}\left(\delta \right)}{\partial C_{m}^{t}}\left|s\right.+\frac{\partial L_{Q}\left(\delta \right)}{\partial C_{m}^{t}}\left|t\right.=\frac{\partial L_{Q}\left(\delta \right)}{\partial U_{m}^{t}}+\eta_{c}\cdot \frac{\partial L_{Q}\left(\delta \right)}{\partial C_{m}^{t+1}},\;\;\;t<T,m<M \end{array}\right.$$

where |s and |t denote derivatives in the spatial domain (along network layers) and temporal domain (along time steps), respectively; $O_{M}^{t}$ is the spike output of the *m*-*th* layer at time step t; and $\dot{T}\left(\cdot \right)$ represents the pseudo-derivative of the spike-generation function *T*(·).

## 4. Experimental Discussion and Analysis

### 4.1. Experimental Setup

**Figure 9:**
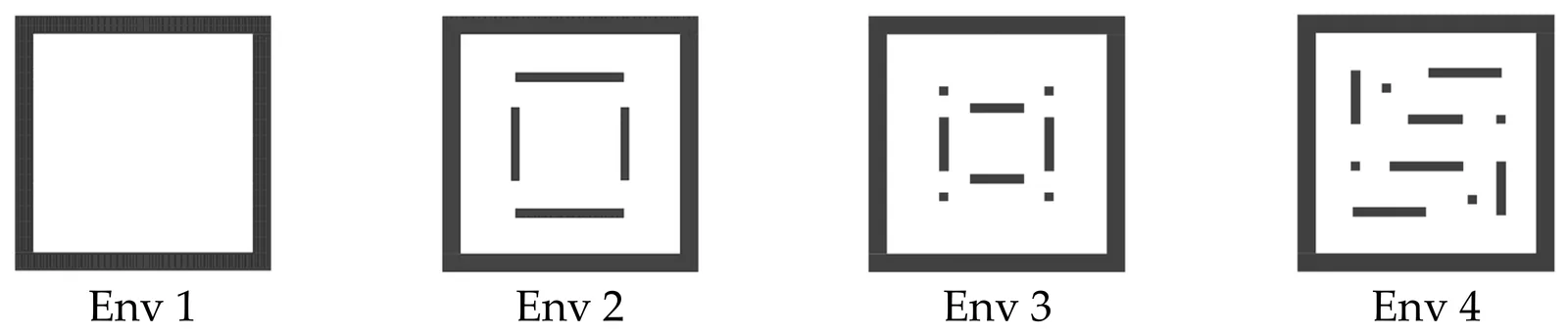
Schematic of the training environment.

The training and evaluation environments are constructed based on the robot operating system (ROS). A curriculum learning approach is adopted during the training process, progressing from simple to complex scenarios across four distinct training environments (as shown in Figure 9). The training environment has dimensions of 20 m × 20 m. Two evaluation environments with different complexity levels are also established to evaluate the generalization capability of the proposed algorithm. The detailed experimental configurations and network hyperparameters are listed in Table 1 and Table 2, respectively.

### 4.2. Performance Analysis of Training Process

To validate the proposed NSM-CBAM, three representative baselines are selected based on the incremental components of the proposed method. It should be noted that all methods compared in this work, including the spiking methods, operate in the same continuous action space. For spiking methods, the output spike train is averaged over the time step *T* to obtain action probabilities in [0, 1], which are then mapped to continuous linear and angular velocity commands. Therefore, the comparison is conducted under identical action dimensions and constraints. DDPG is a standard continuous-control DRL baseline without spiking neurons or explicit memory. SDDPG introduces spiking neurons into DDPG, thereby allowing the contribution of spiking computation to be evaluated. Spiking-LSTM further introduces a spiking LSTM memory structure, thereby allowing the contribution of memory to be evaluated. The proposed NSM-CBAM adds the spiking memory CBAM module on top of this structure, thereby introducing channel and spatial attention into the memory. Therefore, the comparison follows a controlled incremental design. This design isolates the effects of spiking computation, memory, and attention. Other spiking alternatives are not directly used as baselines because they involve different action encodings, problem settings, or training paradigms, which would introduce additional variables and make a controlled comparison difficult. Other methods reviewed in the Introduction, including TD3 and PPO, are regarded as related work rather than experimental baselines, as this work focuses on component-wise validation rather than exhaustive benchmarking.

**Figure 10:**
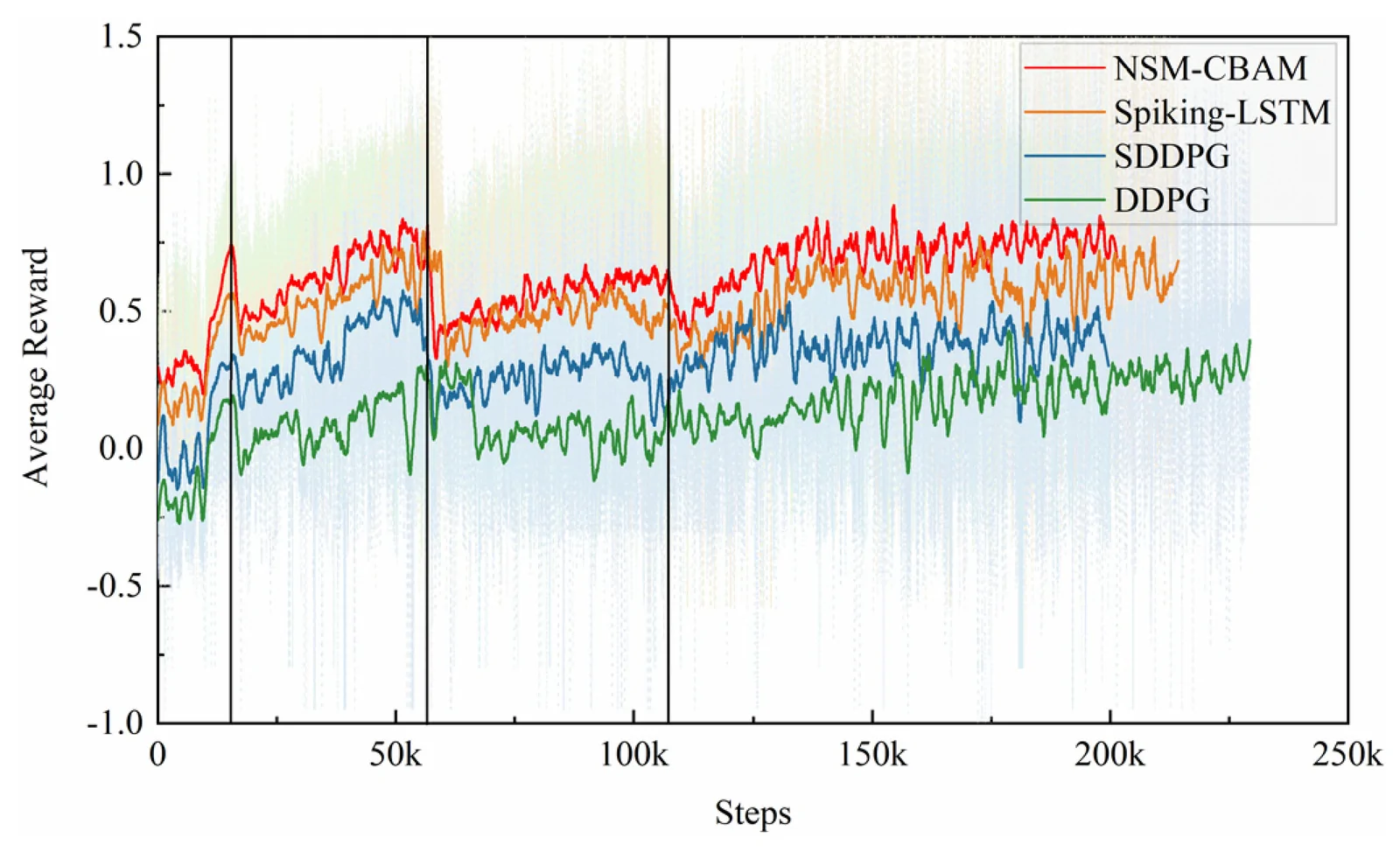
Average reward curves of different methods in four training environments (*T* = 5).

**Figure 11:**
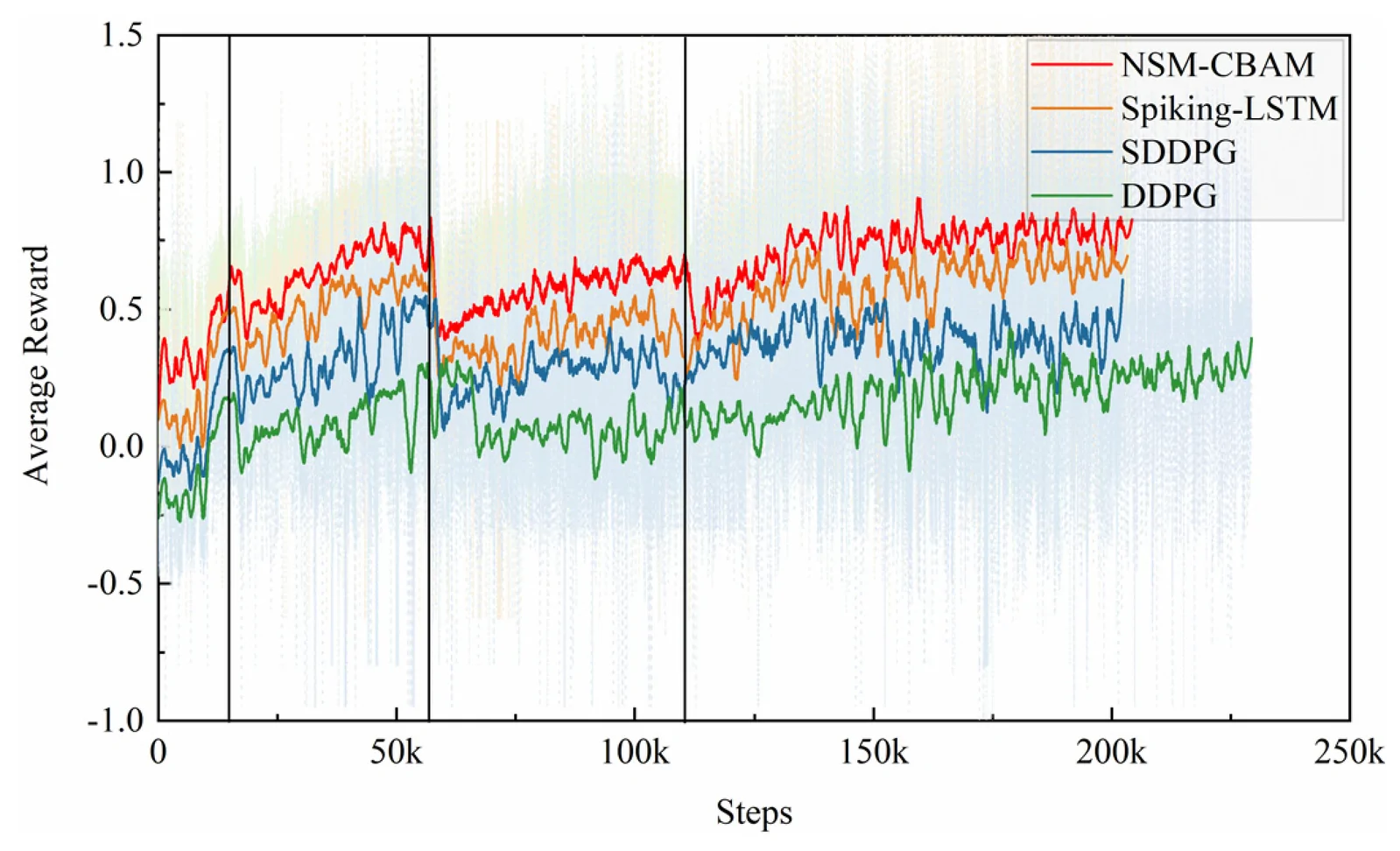
Average reward curves of different methods in four training environments (*T* = 10).

**Figure 12:**
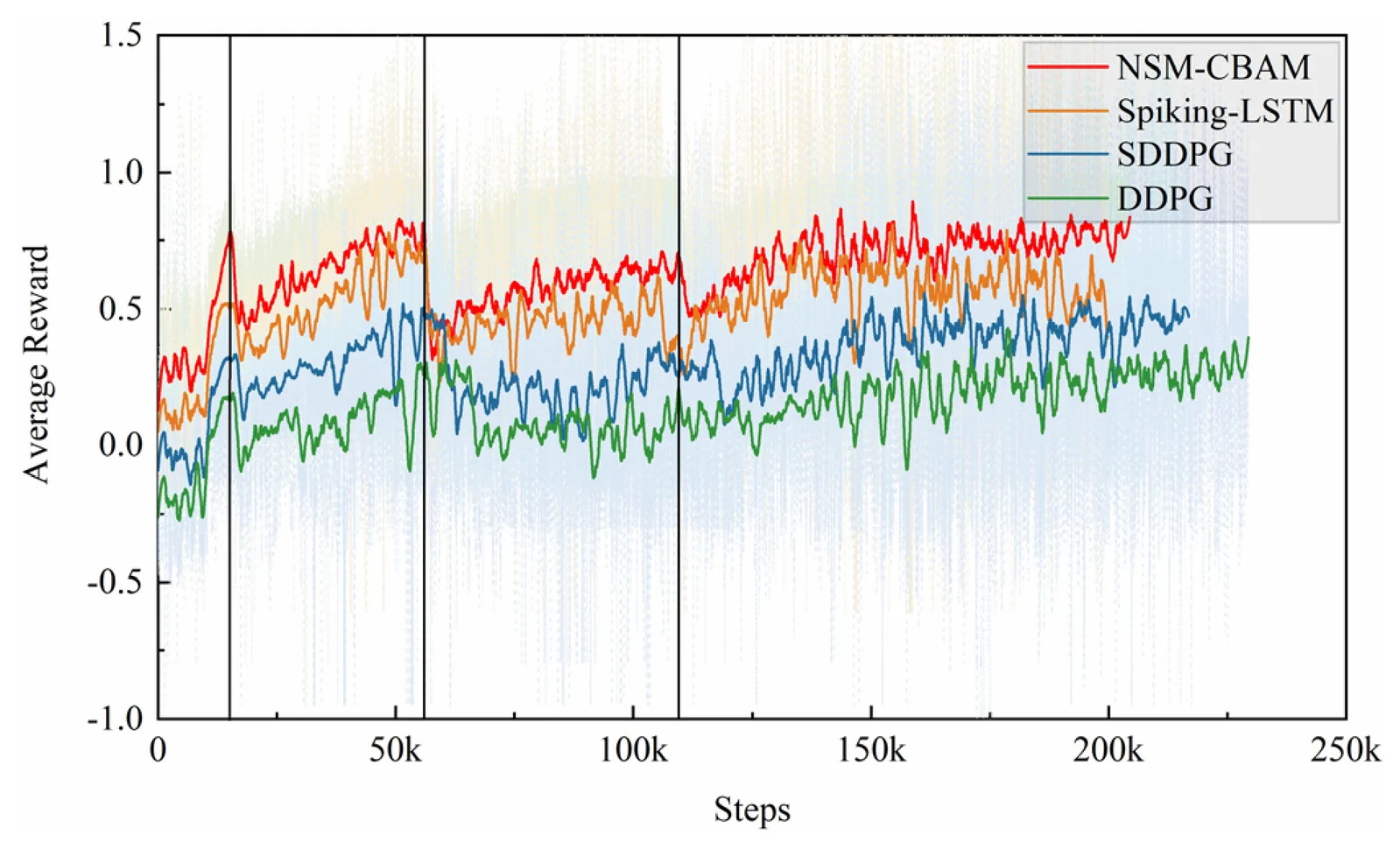
Average reward curves of different methods in four training environments (*T* = 15).

The variation of the average reward across four different training environments is analyzed, and the impact of different time step lengths *T* on the learning of autonomous collision avoidance strategies for UAVs has been further investigated. The training results are shown in Figure 10, Figure 11 and Figure 12. During training, 100, 200, 300, and 400 start-goal point pairs are used for training Env 1, training Env 2, training Env 3, and training Env 4, respectively, with coordinates randomly initialized. Figure 10 presents the average reward curves for the four collision avoidance strategies in training Env 1–4 when *T* = 5. As environmental difficulties increase, all four strategies require more training.

However, except for the proposed NSM-CBAM model, the other three comparison methods have exhibited significant fluctuations in the later stages of training, particularly the DDPG method, which shows the most severe oscillations. This indicates a lack of stability and robustness in these comparison methods, as they fail to learn a stable collision avoidance policy effectively. As the forward propagation time T of the spiking neurons increases to *T* = 10 and *T* = 15 (Figure 11 and Figure 12), the longer propagation time improves the response capability of the neurons and thereby enhances the stability and robustness of Spiking-LSTM and SDDPG to some extent. This reduces fluctuations in the average reward curves and improves the collision avoidance performance of the UAV. Nevertheless, compared with the NSM-CBAM model, Spiking-LSTM and SDDPG still show relatively large fluctuations and did not enable the UAV to learn autonomous collision avoidance behavior as stably or efficiently in complex environments.

**Figure 13:**
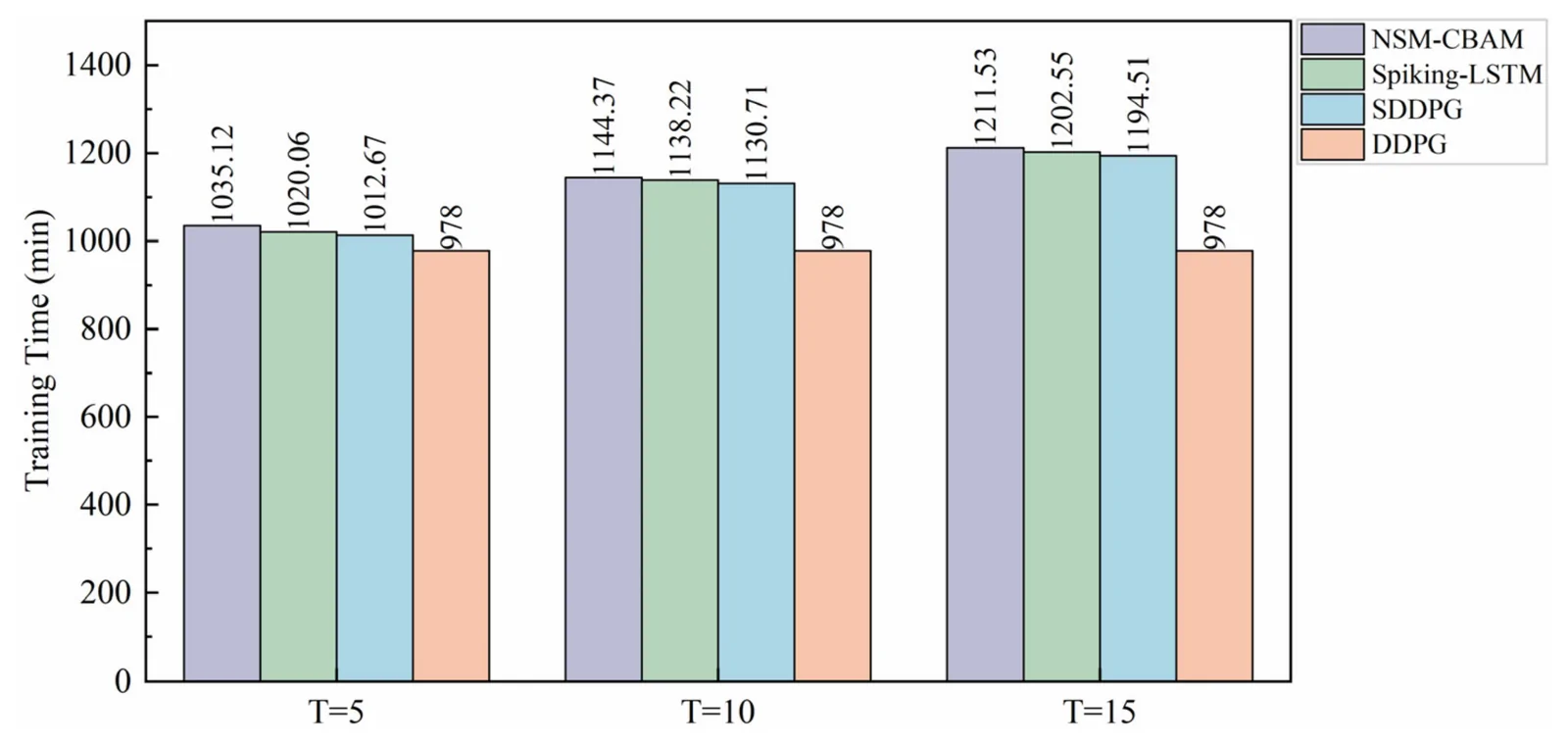
Training time of different collision avoidance methods at different time steps.

Additionally, a comparative analysis of the training times for different collision avoidance methods under three distinct time steps (*T* = 5, *T* = 10, *T* = 15) is conducted. The results are shown in Figure 13. DDPG has the shortest total training time. As *T* increases, the training times of NSM-CBAM, Spiking-LSTM, and SDDPG all increase. Across *T* = 5, 10, and 15, SDDPG consistently trains faster than NSM-CBAM and Spiking-LSTM. However, despite its faster training, SDDPG has failed to learn an effective collision avoidance policy, yielding lower success rates and poor generalization in unknown evaluation environments. By contrast, although the proposed NSM-CBAM has a relatively longer training time, its spiking memory CBAM module effectively captures important historical information from spatial and temporal channels. By leveraging historical memory and temporal continuity, it produces more accurate collision avoidance decisions and improved success rates. Furthermore, to avoid the computational resource consumption and slower convergence associated with prolonged training, *T* = 5 is selected as the final time step parameter. This choice balances training performance with computational cost while ensuring satisfactory accuracy and generalization ability, thereby ensuring that the UAV can learn an effective collision avoidance policy within a reasonably short training duration.

### 4.3. Ablation Study

To evaluate the effectiveness of the spiking memory CBAM module within the proposed NSM-CBAM model, an ablation study has been conducted. Table 3 presents the experimental results in two evaluation environments. Baseline denotes the standard SDDPG model without any memory structure. Spiking-LSTM refers to the addition of a spiking LSTM structure to the baseline model. Spiking channel LSTM refers to the model that incorporates only the channel attention mechanism based on the baseline, and spiking spatial LSTM represents the model that incorporates only the spatial attention mechanism based on the baseline.

As shown in Table 3, compared to the baseline, the integration of the proposed spiking memory CBAM module improves the collision avoidance success rate by 5% and 5.5% in the two evaluation environments, respectively. This improvement exceeds that of the other three ablation configurations, indicating that adding only a memory structure or only an attention mechanism is insufficient to capture critical historical information in complex environments and still carries a nonnegligible risk of avoidance failure. Furthermore, compared with the hard-reset neuron mechanism, the soft-reset neuron mechanism employed in this study achieves a higher success rate in UAV obstacle avoidance. During autonomous collision avoidance, the model must both perceive current environmental features and incorporate state information from the previous time step. By combining the dual mechanisms of channel and spatial attention, the spiking memory CBAM module focuses on key information relevant to the collision avoidance task within complex settings. It adaptively recalls historical information from both spatial and temporal channels to optimize avoidance decisions, thereby enabling more precise path planning and policy adjustments. The experimental results have shown that the NSM-CBAM model, equipped with the spiking memory CBAM module, has stronger spatio-temporal feature perception and greater environmental adaptability. While maintaining flight safety, it substantially improves the autonomous collision avoidance performance of the UAV.

### 4.4. Performance Analysis of Evaluation Process

To validate the collision avoidance performance of the proposed NSM-CBAM decision-making method, we have used the success rate and collision rate as evaluation metrics to assess the autonomous collision avoidance capabilities of the proposed model and the comparison methods. The success rate denotes the percentage of evaluation episodes in which the UAV reached the target point without any collision out of the total number of evaluations. The collision rate denotes the percentage of evaluation episodes in which the UAV collided with an obstacle, resulting in task failure. During the evaluation, five independent runs were conducted, with 200 pairs of starting and goal points randomly generated in each run. The results were averaged over the five runs to reduce the influence of randomness. The comparative results for evaluation Env 1 and evaluation Env 2 are presented in Table 4. As a policy-gradient reinforcement learning method, DDPG updates its policy using deterministic action selection, which makes it prone to converging to local optima in complex environments. Consequently, DDPG performs poorly in collision avoidance evaluation, achieving success rates of only 81.5% and 79.5%. Both TD3 and PPO achieve higher success rates than DDPG. However, due to their limited ability to retain information from historical states, their success rates remain lower than that of the proposed method.

By contrast, SDDPG incorporates SNN, whose spatio-temporal processing and computation more closely resemble biological neurons. SDDPG has achieved higher success rates of 89% and 87%, clearly outperforming DDPG in collision avoidance. Building upon this, the memory structure added in Spiking-LSTM enhances the model’s memory of historical information. It allows the model to maintain stable state updates and memory retention during collision avoidance tasks, thereby further improving its success rate. Compared with the aforementioned models, the proposed NSM-CBAM incorporates a spiking memory CBAM module that adaptively assigns weights to historical information via channel and spatial attention mechanisms. This module efficiently extracts task-relevant features in complex environments, integrates information across spatial and temporal dimensions, increases the use of important historical information, and leverages memory to update avoidance strategies promptly.

As a result, it produces more efficient and precise collision avoidance decisions in complex settings. The NSM-CBAM model has achieved the highest success rates in evaluation Env 1 and evaluation Env 2, reaching 94% and 92.5%, respectively, and outperformed all other comparison methods. Therefore, the NSM-CBAM method demonstrates adaptive collision avoidance capabilities, with generalization and stability in unseen environments containing obstacle shapes and densities outside the training distribution.

Table 5 compares the average path length and average navigation time for each method in evaluation Env 1 and evaluation Env 2. The results have shown that, while maintaining a high collision avoidance success rate for the UAV, the proposed method in this paper has achieved competitive performance in both path length and navigation time. The results demonstrate that the proposed method can achieve more efficient trajectory planning while ensuring flight safety and avoiding collisions. It has successfully avoided obstacles while effectively reducing redundant detours, which exhibits effective autonomous collision avoidance capabilities.

**Figure 14:**
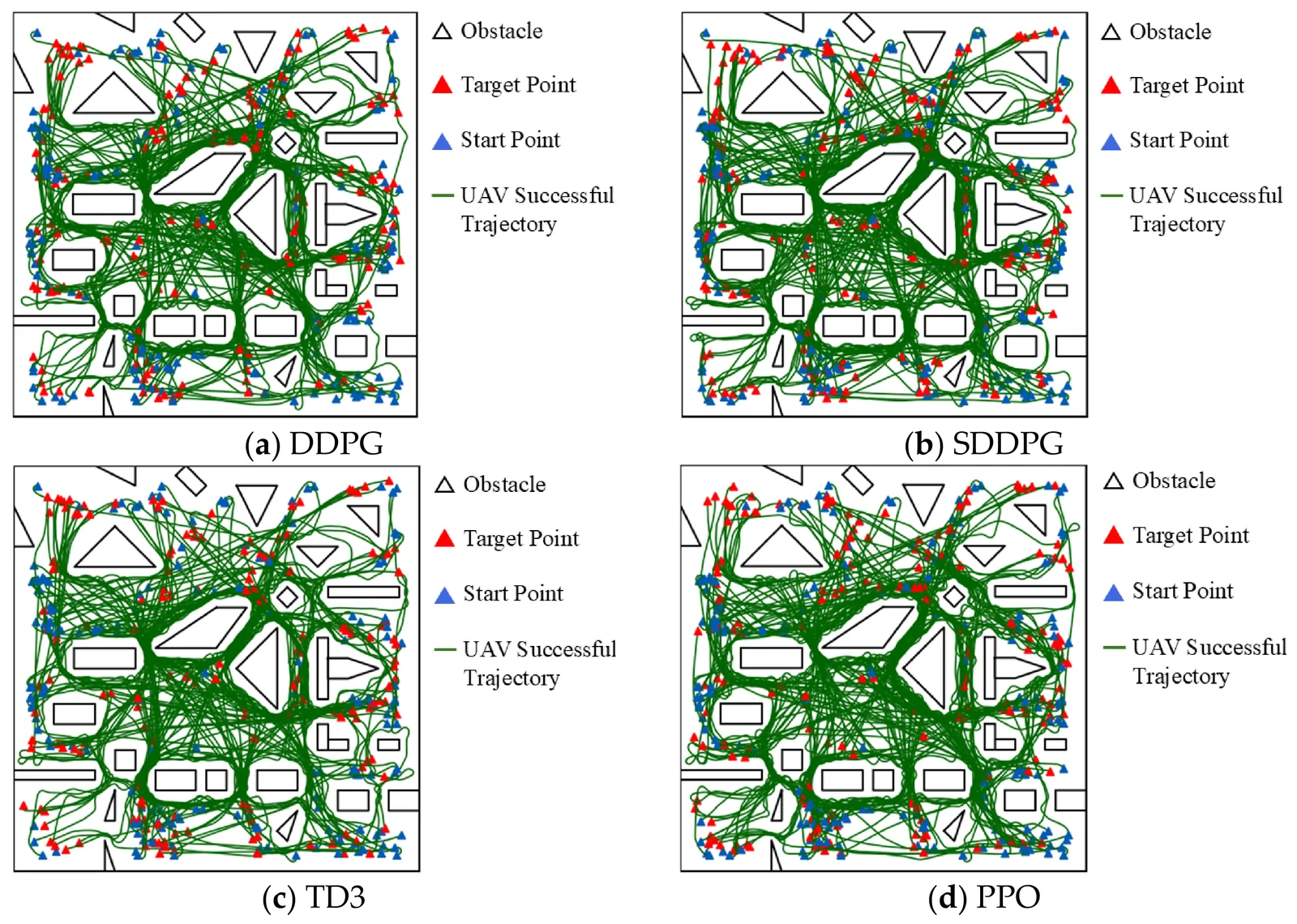

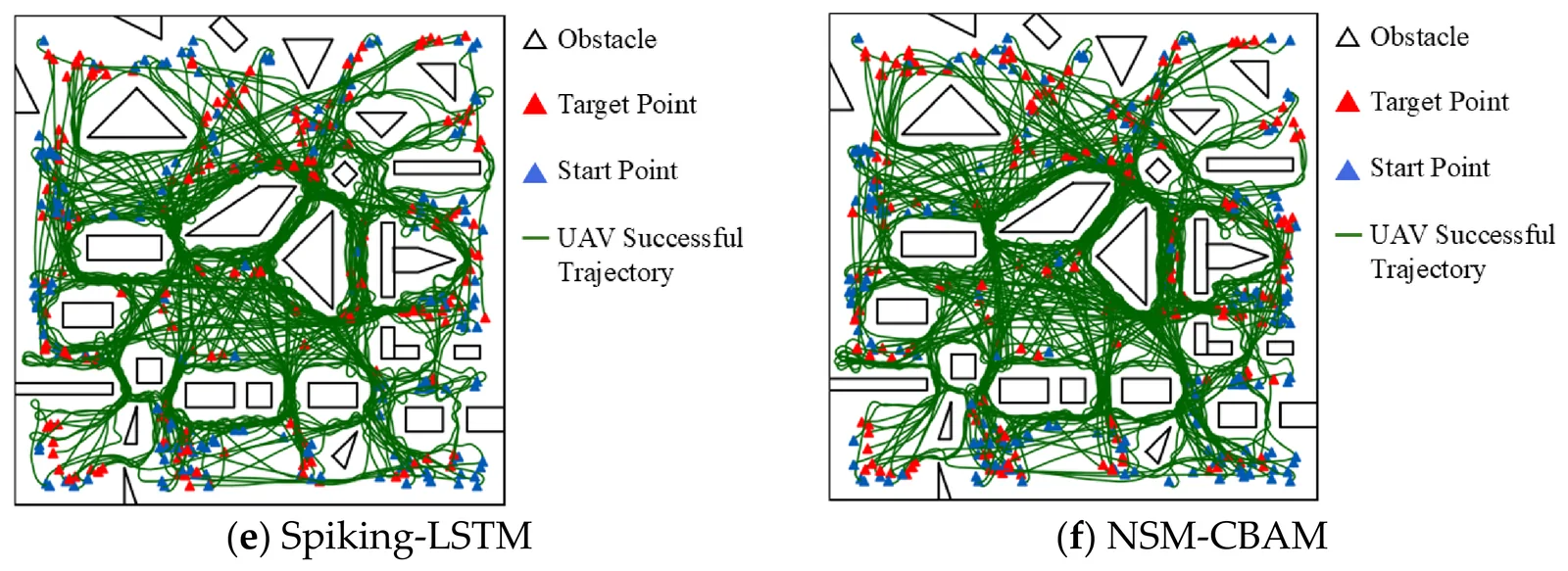
Successful trajectories of different methods (Evaluation Env 1, 20 m × 20 m): (**a**) DDPG. (**b**) SDDPG. (**c**) TD3. (**d**) PPO. (**e**) Spiking-LSTM. (**f**) NSM-CBAM.

**Figure 15:**
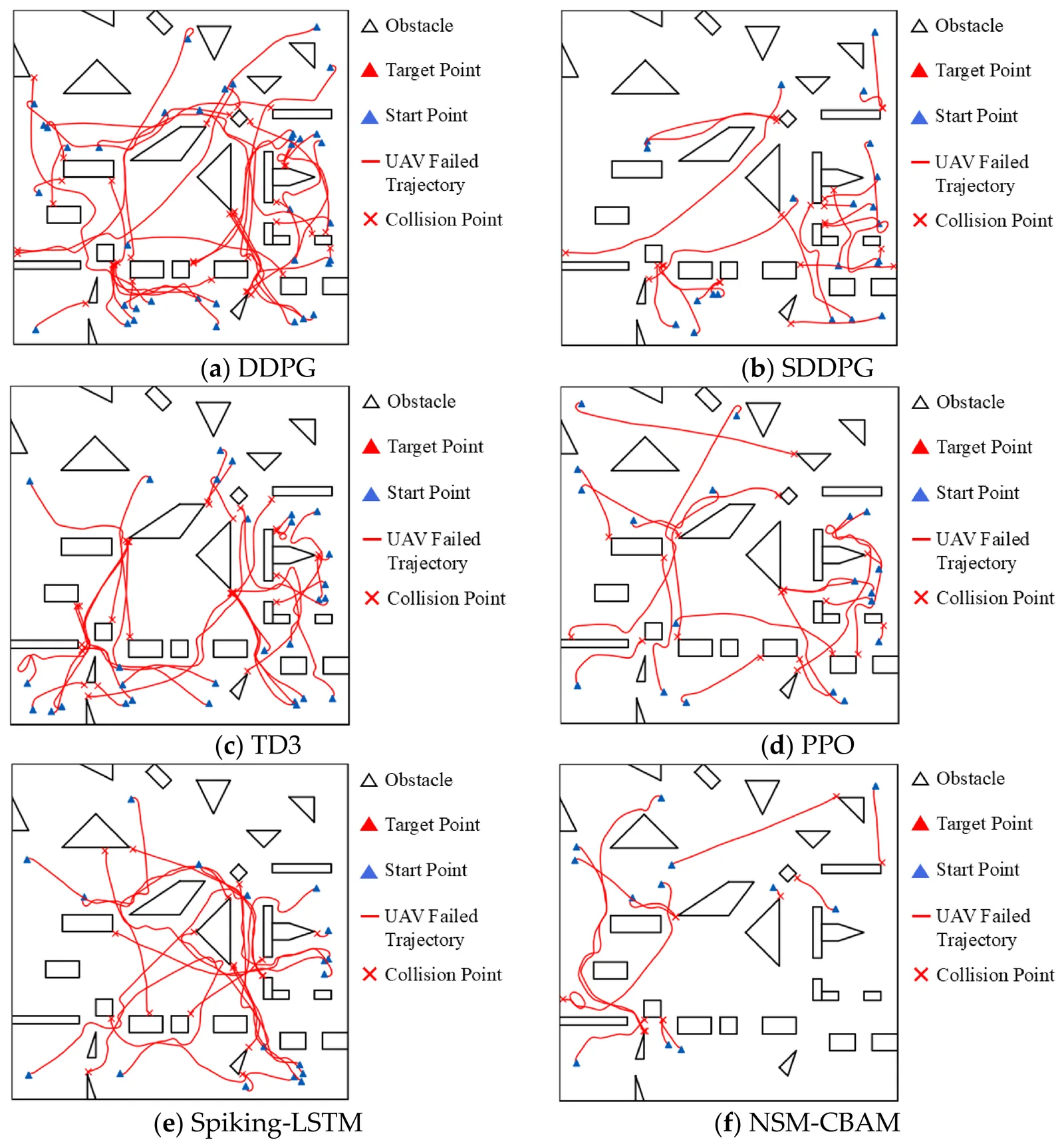
Failure trajectories of different methods (Evaluation Env 1, 20 m × 20 m): (**a**) DDPG. (**b**) SDDPG. (**c**) TD3. (**d**) PPO. (**e**) Spiking-LSTM. (**f**) NSM-CBAM.

**Figure 16:**
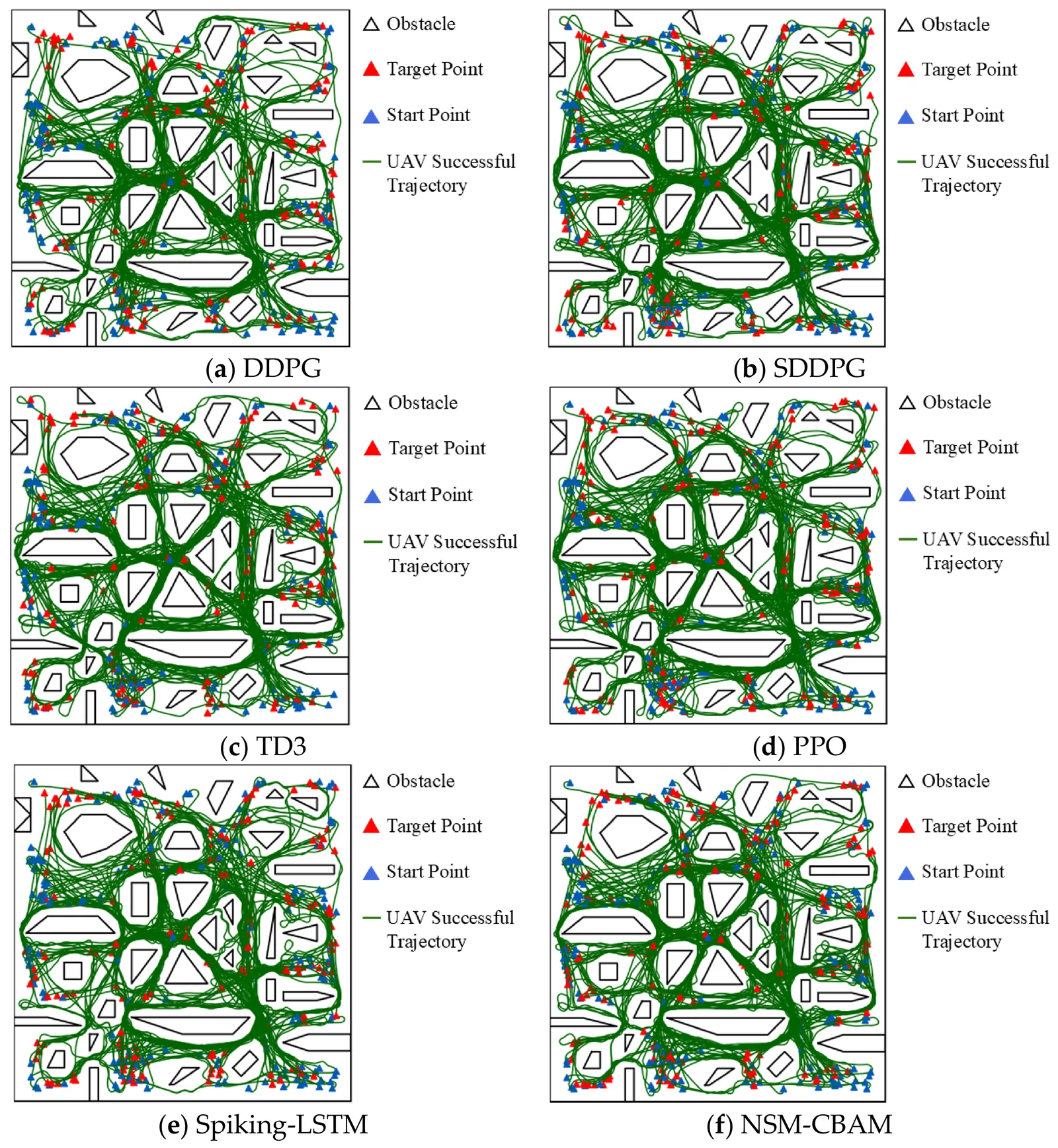
Successful trajectories of different methods (Evaluation Env 2, 20 m × 20 m): (**a**) DDPG. (**b**) SDDPG. (**c**) TD3. (**d**) PPO. (**e**) Spiking-LSTM. (**f**) NSM-CBAM.

**Figure 17:**
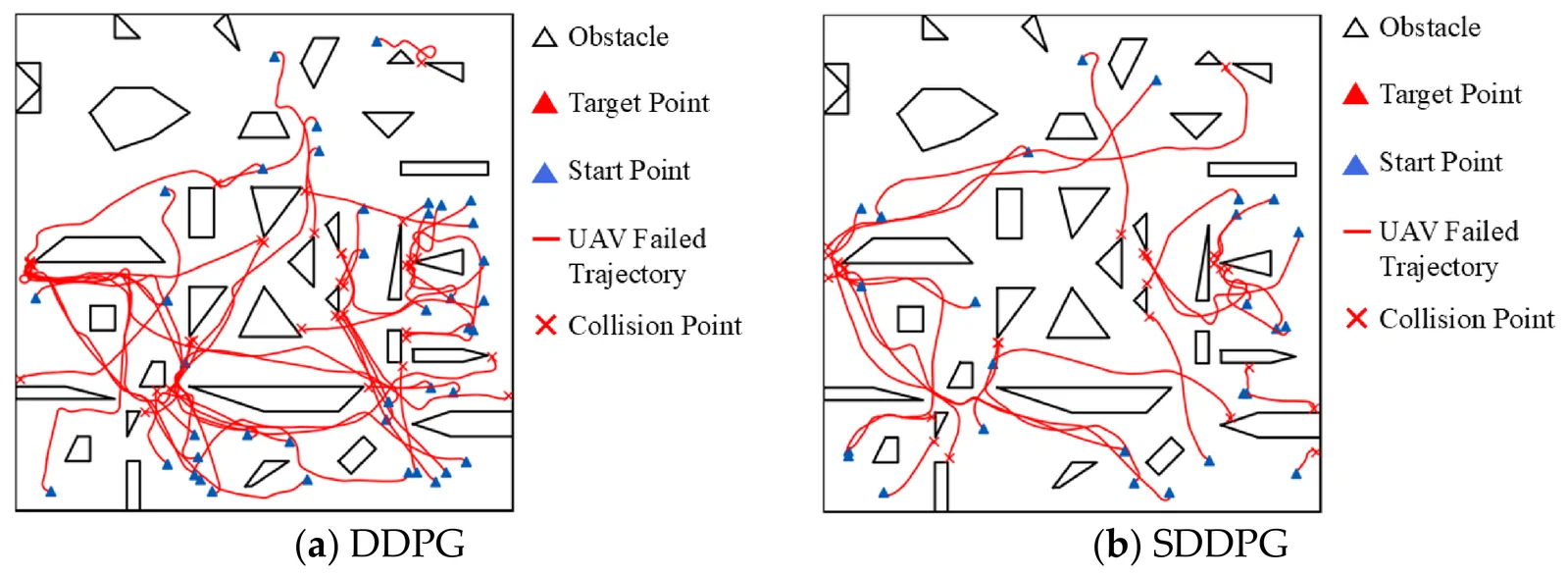

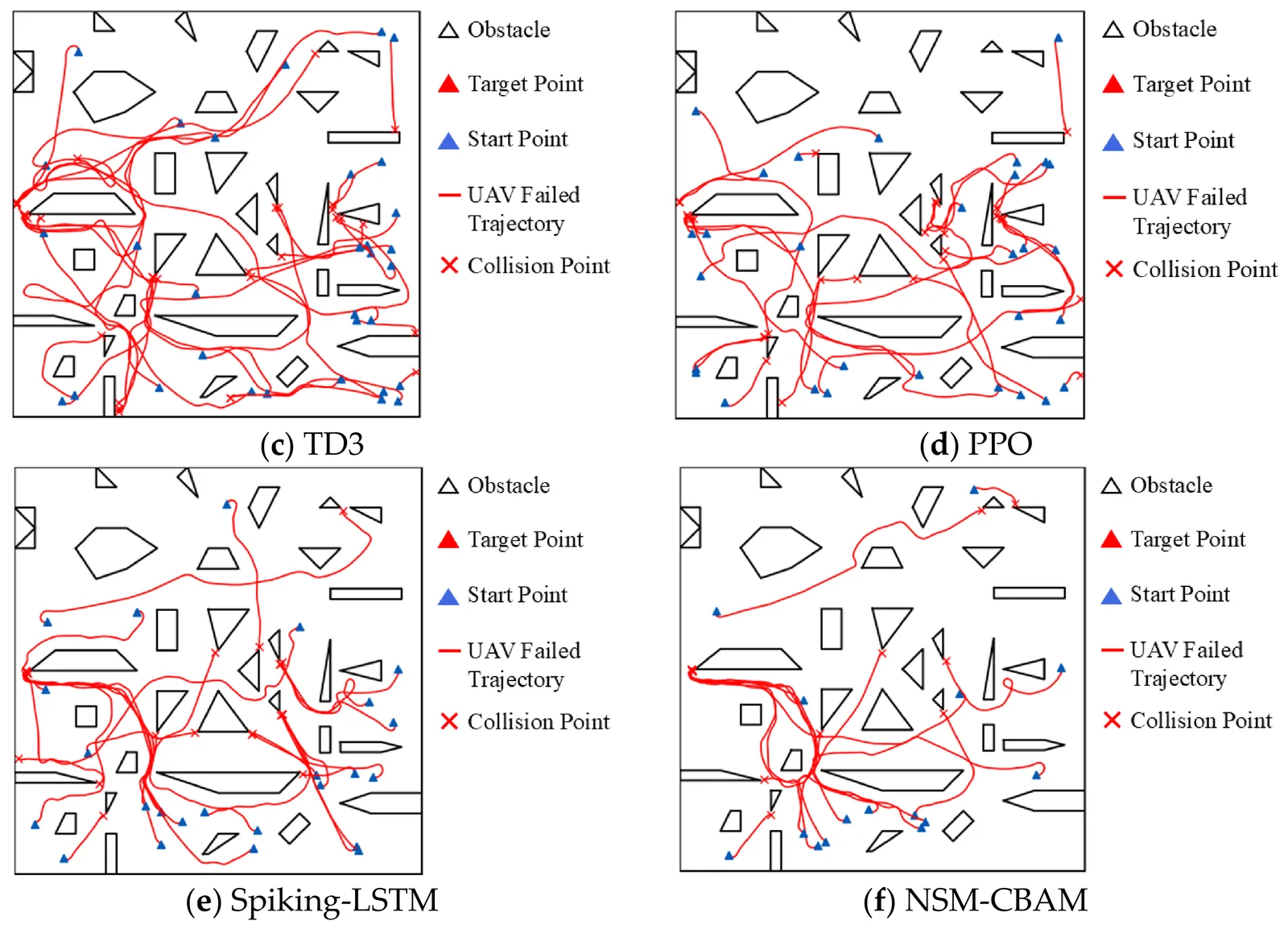
Failure trajectories of different methods (Evaluation Env 2, 20 m × 20 m): (**a**) DDPG. (**b**) SDDPG. (**c**) TD3. (**d**) PPO. (**e**) Spiking-LSTM. (**f**) NSM-CBAM.

The successful and failure trajectories of the proposed NSM-CBAM and other known methods in evaluation Env 1 and evaluation Env 2 are shown in Figure 14, Figure 15, Figure 16 and Figure 17. Specifically, Figure 14 and Figure 16 present successful cases, while Figure 15 and Figure 17 present failures. The blue triangle represents the start point, and the red triangle denotes the target point. The red cross represents the collision point. The green line represents the successful trajectory, and the red line denotes the failure trajectory. The trajectory distributions indicate that DDPG suffers the most collisions, particularly at the sharp corners of obstacles. This is because DDPG, as an ANN-based method, lacks the ability for spatio-temporal information processing, which makes it difficult to make correct collision avoidance decisions promptly in complex environments, thereby affecting its performance.

**Figure 18:**
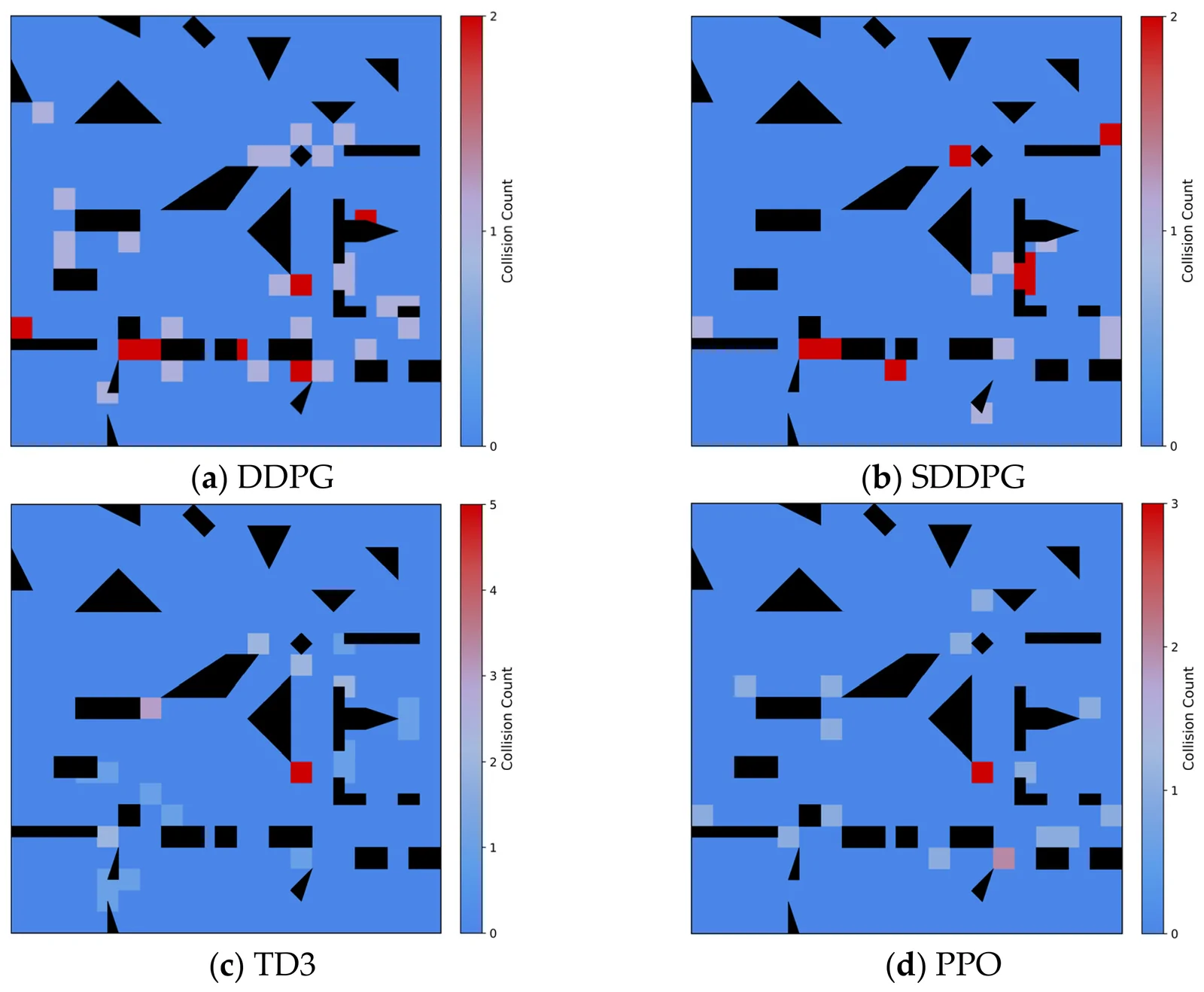

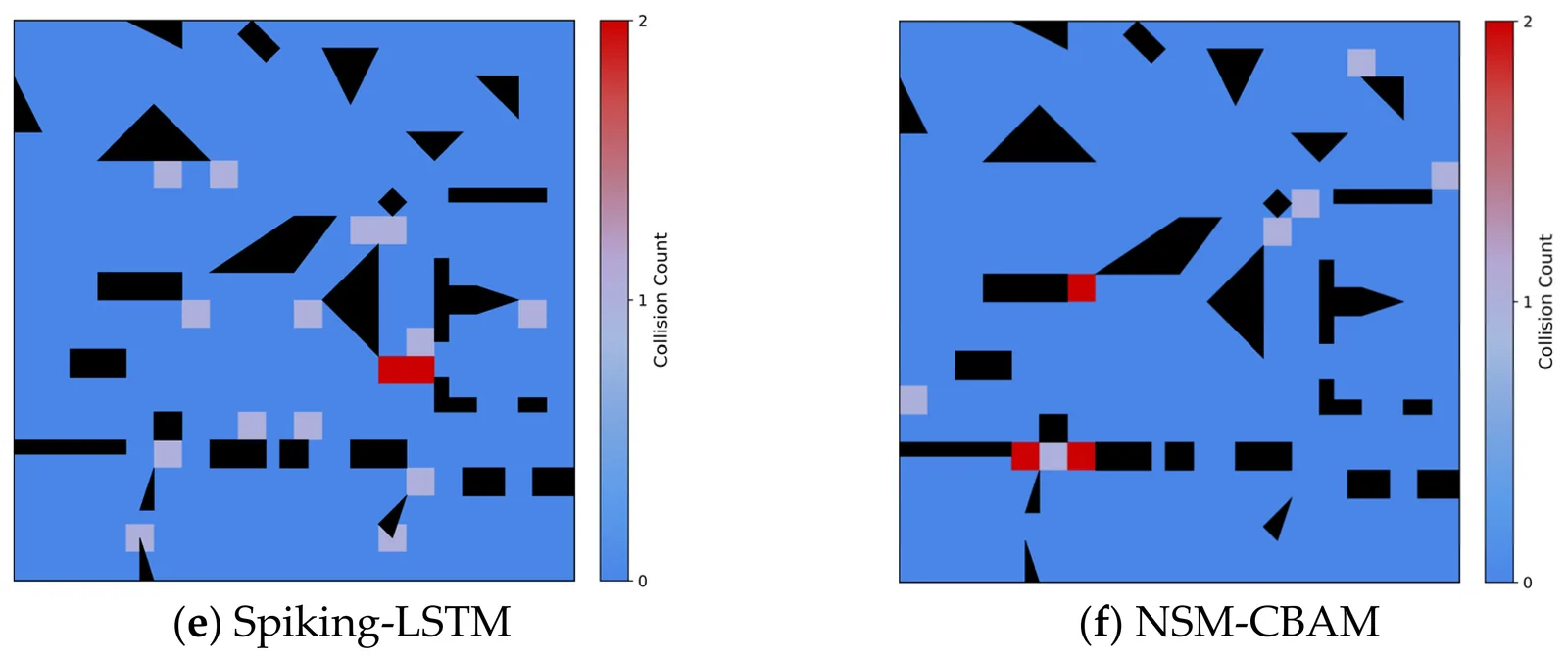
Collision heatmap (Evaluation Env 1, 20 m × 20 m): (**a**) DDPG. (**b**) SDDPG. (**c**) TD3. (**d**) PPO. (**e**) Spiking-LSTM. (**f**) NSM-CBAM.

**Figure 19:**
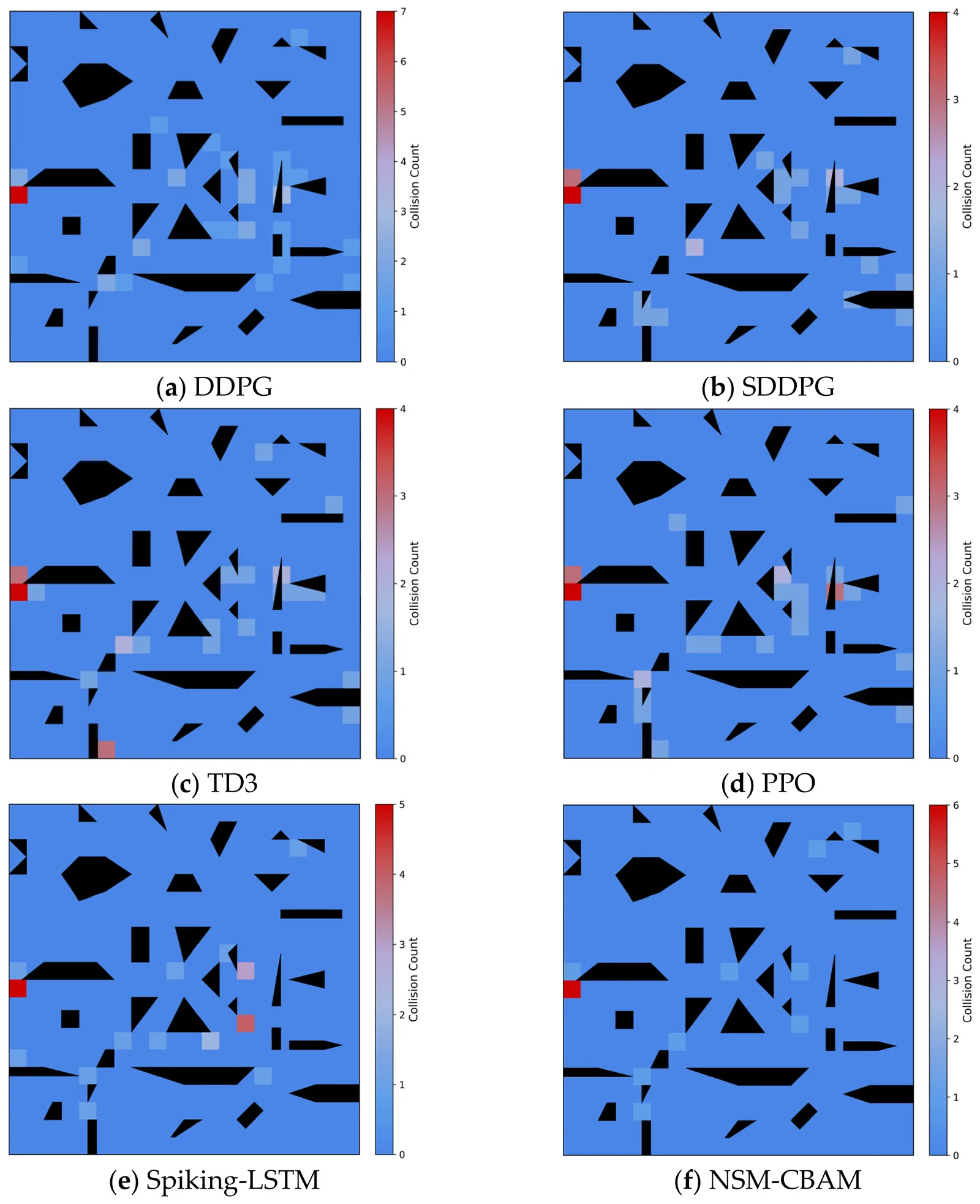
Collision heatmap (Evaluation Env 2, 20 m × 20 m): (**a**) DDPG. (**b**) SDDPG. (**c**) TD3. (**d**) PPO. (**e**) Spiking-LSTM. (**f**) NSM-CBAM.

Since SDDPG adopts the SNN structure, it possesses spatio-temporal information processing ability. Spiking-LSTM utilizes a gate mechanism to dynamically adjust the updating and forgetting of historical memories. Although these two methods can improve collision avoidance success rates to a certain extent, they are still not optimal in performance. In contrast, the proposed NSM-CBAM incorporates a spiking memory CBAM module that combines channel attention and spatial attention mechanisms. It can efficiently integrate spatial and temporal information, which has enhanced the representation of critical historical data and facilitates prompt modification of collision avoidance strategies. Consequently, the proposed NSM-CBAM has demonstrated competitive performance in the autonomous collision avoidance task for UAVs. It achieves shorter flight trajectories, less time consumption, and the highest collision avoidance success rate within the evaluated environments, while exhibiting adaptability and generalization capabilities. These results suggest potential for enhancing the autonomous collision avoidance performance of UAVs in more complex environments. Figure 18 and Figure 19 present the collision heatmaps for two different test scenarios. It can be observed that collisions occur more frequently near sharp corners of obstacles and in densely cluttered regions. By incorporating memory of historical states, the proposed method can effectively reduce the number of collisions in these high-risk regions compared with the baseline methods.

In this study, a theoretical estimation is provided to verify the energy efficiency of the proposed method compared with an ANN with the same network architecture. Such theoretical analyses have been widely employed to compare the computational energy consumption of SNNs and ANNs under identical network architectures [49,50]. According to the computational energy consumption benchmarks for 45 nm CMOS operations [51], a conventional ANN requires one floating-point addition and one floating-point multiplication to perform a multiply–accumulate (MAC) operation. In contrast, owing to the event-driven nature of SNNs, computations are triggered only when spiking neurons generate spikes after their membrane potentials exceed the firing threshold, thereby reducing unnecessary computational operations and the associated energy consumption. Moreover, due to binary spike-based information transmission, synaptic computations in SNNs can be implemented using accumulation (AC) operations instead of MAC operations. Accordingly, the computational energy consumption of the ANN and SNN can be estimated using the following equations:

(39) $$E_{\mathrm{ANN}}=\sum_{n}\mathrm{FLO}P_{\mathrm{ANN}}\left(n\right)\times E_{\mathrm{MAC}}$$

(40) $$E_{\mathrm{SNN}}=\sum_{n}\mathrm{FLO}P_{\mathrm{SNN}}\left(n\right)\times E_{\mathrm{AC}}$$

It can be observed that the ratio of the computational energy consumption of the proposed method to that of an ANN with the same network architecture, denoted by *E_SNN_*_/*ANN*_ = *E_NSM_*_−*CBAM*_/*E_ANN_*, is approximately 10.2%. In other words, the spiking policy network consumes only approximately 10.2% of the computational energy required by an ANN with an identical architecture. This result demonstrates the computational energy-efficiency advantage of the proposed method.

## 5. Conclusions

In this paper, we propose an NSM-CBAM framework with a spiking attention mechanism for autonomous collision avoidance by UAVs in complex and unknown environments. The framework is based on an actor-critic structure, consisting of SMCAN and DCN. Spiking memory CBAM is introduced into SMCAN to extract key historical states in spatial and temporal channels, which effectively enhances the utilization efficiency of historical memory while suppressing redundant information. The SR-LIF neuron is employed to simulate the dynamic changes in the membrane potential of biological neurons. A spiking encoder and a spiking decoder are designed to achieve the conversion between continuous state information and spiking sequences. The training experiment specifically analyzes the convergence and the time required under different training environments and different time steps *T*. Ablation studies and comparative experiments have been utilized to further validate the effectiveness of the spiking memory CBAM, which enhances the performance of collision avoidance. The proposed NSM-CBAM has achieved the highest collision avoidance success rates of 94% and 92.5% in two unknown evaluation environments. Compared to other methods, NSM-CBAM has also used less navigation time and a shorter path distance. It has been demonstrated that the proposed framework can effectively enhance the memory and utilization of critical historical states by UAVs, improving the intelligence level and execution efficiency.

Although the proposed NSM-CBAM achieves promising results in simulated static environments, its performance in dynamic scenarios has not yet been evaluated. Dynamic obstacles introduce time-varying state transitions and require the policy to anticipate future obstacle motion. Such scenarios may involve different state representations, reward designs, and training curricula. The spiking memory CBAM and attention mechanism are designed to capture temporal dependencies and focus on critical historical states, which may be beneficial for dynamic situations, but this remains to be verified. In future work, we will expand this research in two aspects. On the one hand, we will extend the research scenarios from static obstacles to dynamic ones, creating more complex environments to better simulate real-world conditions and further enhancing the response and collision avoidance capabilities of the proposed framework against rapidly moving obstacles. On the other hand, we plan to deploy the proposed framework onto neuromorphic hardware chips such as Loihi and conduct physical experiments in real environments. By exploiting the event-driven and low-energy properties of such chips, we will construct an efficient, energy-efficient, and bio-inspired autonomous collision avoidance system to support the long-term autonomous operation of UAVs in natural environments.

## Figures and Tables

**Table 1:** Specific experimental configurations.

Name of Software and Hardware	Specific Model and Parameters
Operation System	Ubuntu 18.04
CPU	Intel Core I9-14900KF
GPU	NVIDIA GeForce RTX 4090
Simulation System	ROS Melodic
Simulator	Gazebo
Development Languages	Python 3.5.2 and C++ 11
Training Framework	Pytorch-GPU
Integrated Development Environment	VSCode

**Table 2:** Hyperparameters for NSM-CBAM.

Hyperparameters	Values
Number of neurons per hidden layer in SMCAN	512
Number of neurons per hidden layer in DCN	512
Learning rate of SMCAN	0.00001
Learning rate of DCN	0.0001
Buffer capacity, batch size	100,000, 256
Exploration mechanism	Ornstein–Uhlenbeck (OU) noise
Soft update factor, discount factor	0.01, 0.999
OU parameters (*θ*, *μ*, *σ*)	*θ* = 0.15, *μ* = 0, *σ*: 0.2–0.02 (linear decay)
Reward thresholds (goal_th, obs_th)	0.35, 0.3
Time steps *T*	5, 10, 15
Decay factor of spiking neuron *η_v_*, *η_c_*, *U*_th_	0.75, 0.5, 0.5

**Table 3:** Ablation studies with different methods in different evaluation environments.

Method	Env 1 (Success Rate)	Env 2 (Success Rate)
Baseline	89%	87%
Spiking-LSTM	91%	88%
Spiking Channel LSTM	90.5%	89%
Spiking Spatial LSTM	91.5%	89.5%
NSM-CBAM (LIF)	92%	90%
NSM-CBAM	94%	92.5%

**Table 4:** Comparative experiment results with known methods.

Method	Env 1 (Success Rate)	Env 2 (Success Rate)
DDPG	81.5%	79.5%
SDDPG	89%	87%
TD3	85.5%	84%
PPO	90.5%	85%
Spiking-LSTM	91%	88%
NSM-CBAM	94%	92.5%

**Table 5:** Average path distance and average path time with known methods.

Evaluation Environment	Metrics	DDPG	SDDPG	TD3	PPO	Spiking-LSTM	NSM-CBAM
Env 1	Average Path Distance (m)	16.99	17.11	16.78	17.27	16.87	16.56
	Average Path Time (s)	37.74	38.05	37.85	37.98	37.30	37.04
Env 2	Average Path Distance (m)	18.40	18.45	18.34	18.38	17.63	17.41
	Average Path Time (s)	40.46	40.36	40.45	40.26	39.91	38.88

## Data Availability

The original contributions presented in this study are included in the article. Further inquiries can be directed to the corresponding author.
